# Antithetic effect of interferon-α on cell-free and cell-to-cell HIV-1 infection

**DOI:** 10.1371/journal.pcbi.1010053

**Published:** 2022-04-25

**Authors:** Ryuichi Kumata, Shoya Iwanami, Katrina B. Mar, Yusuke Kakizoe, Naoko Misawa, Shinji Nakaoka, Yoshio Koyanagi, Alan S. Perelson, John W. Schoggins, Shingo Iwami, Kei Sato

**Affiliations:** 1 Division of Systems Virology, Department of Infectious Disease Control, International Research Center for Infectious Diseases, Institute of Medical Science, The University of Tokyo, Tokyo, Japan; 2 Laboratory of Systems Virology, Institute for Frontier Life and Medical Sciences, Kyoto University, Kyoto, Japan; 3 Faculty of Science, Kyoto University, Kyoto, Japan; 4 interdisciplinary Biology Laboratory (iBLab), Division of Biological Science, Graduate School of Science, Nagoya University, Nagoya, Japan; 5 Department of Microbiology, University of Texas Southwestern Medical Center, Dallas, Texas, United States of America; 6 Mathematical Biology Laboratory, Department of Biology, Faculty of Sciences, Kyushu University, Fukuoka, Japan; 7 Laboratory of Mathematical Biology, Faculty of Advanced Life Science, Hokkaido University, Sapporo, Japan; 8 PRESTO, Japan Science and Technology Agency, Saitama, Japan; 9 Theoretical Biology and Biophysics, Los Alamos National Laboratory, Los Alamos, New Mexico, United States of America; 10 CREST, Japan Science and Technology Agency, Saitama, Japan; 11 MIRAI, Japan Science and Technology Agency, Saitama, Japan; 12 Institute for the Advanced Study of Human Biology (ASHBi), Kyoto University, Kyoto, Japan; 13 NEXT-Ganken Program, Japanese Foundation for Cancer Research (JFCR), Tokyo, Japan; 14 Science Groove Inc., Fukuoka, Japan; Johns Hopkins University, UNITED STATES

## Abstract

In HIV-1-infected individuals, transmitted/founder (TF) virus contributes to establish new infection and expands during the acute phase of infection, while chronic control (CC) virus emerges during the chronic phase of infection. TF viruses are more resistant to interferon-alpha (IFN-α)-mediated antiviral effects than CC virus, however, its virological relevance in infected individuals remains unclear. Here we perform an experimental-mathematical investigation and reveal that IFN-α strongly inhibits cell-to-cell infection by CC virus but only weakly affects that by TF virus. Surprisingly, IFN-α enhances cell-free infection of HIV-1, particularly that of CC virus, in a virus-cell density-dependent manner. We further demonstrate that LY6E, an IFN-stimulated gene, can contribute to the density-dependent enhancement of cell-free HIV-1 infection. Altogether, our findings suggest that the major difference between TF and CC viruses can be explained by their resistance to IFN-α-mediated inhibition of cell-to-cell infection and their sensitivity to IFN-α-mediated enhancement of cell-free infection.

## Author summary

HIV-1 experiences a strong bottleneck during transmission, and only the virus(es) with higher resistance to the host’s innate immunity, interferon (IFN), can be successfully transmitted. Because the IFN resistance tends to be disappeared during infection in infected individuals, this phenotype would be crucial for human-to-human transmission. By combining mathematical modeling with well-designed time-series viral infection experiments, we investigated the difference on the IFN resistance of two types of HIV-1, which were respectively isolated at the acute and chronic phases of infection, and classified it into two virus transmission modes, cell-free and cell-to-cell infections. We found that IFN suppresses HIV-1 cell-to-cell infection, but surprisingly, promotes cell-free infection. Moreover, the virus isolated during chronic infection is more sensitive to the IFN-mediated promoting effect than that isolated during acute infection. Our results suggest that HIV-1 selects different strategies to adapt to different host environments. We further provide an insight how viruses evolve to counteract or hijack the host immunity.

## Introduction

In 2017, approximately 2 million individuals were newly infected with HIV-1, and approximately 47% of the new infections were attributed to sexual contacts (http://www.unaids.org/en). Although HIV-1 is sexually transmitted, its transmission efficiency is quite low [1]: only one or a few viral variants in a donor contribute to establishment of a new infection by sexual transmission [2,3]. These observations suggest that tissues such as vaginal and rectal mucosa are a bottleneck and select virus(es) for increased viral transmission fitness [4]. The viruses that initiate new infection and grow to detectable levels have been called transmitted/founder (TF) viruses [3,5,6]. In addition to TF viruses, viruses from the same individuals during the chronic phase of infection have been isolated and have been called chronic control (CC) viruses. A previous study proposed that the bottleneck of heterosexual HIV-1 transmission appears to act at a stochastic level, favoring although not exclusively, more fit viruses [7]. On the other hand, by using these viruses in *in vitro* cell culture systems, some previous studies reported that the virological phenotype, particularly with regard to sensitivity to type I interferon (Type-I IFN) (e.g., IFN-alpha [IFN-α]) is different between TF and CC viruses with TF viruses being more resistant to the antiviral effects mediated by Type-I IFN [5,6,8]. Since TF and CC viruses are isolated in the acute and chronic phases of infection in the same individuals, their phenotypic properties may reflect HIV-1 disease progression *in vivo*.

Type-I IFN including IFN-α is an innate cytokine that is produced by immune cells recognizing exogenous pathogens (reviewed in references [9–11]). Type-I IFN triggers a cellular signaling cascade and up-regulates hundreds of cellular genes, called IFN-stimulated genes (ISGs). It is well known that some ISGs play pivotal roles in restricting HIV-1 replication (reviewed in references [12–14]). In both *in vitro* cell cultures and infected individuals, viruses including HIV-1 have two modes of spread: cell-free infection and cell-to-cell infection (reviewed in references [4,15–18]). Through experimental-mathematical investigations, we have previously shown that cell-to-cell infection is predominant in the human CD4^+^ T-cell culture system and this mode of infection increases viral fitness by 3.9-fold [19]. However, it remains unclear how Type-I IFN affects these two modes of infections.

Here we perform experiments using IFN-α and three strains of HIV-1, a standard laboratory strain (NL4-3) [20] and a pair of TF and CC viruses that were both isolated from the same infected individual [3,6]. By applying mathematical models to analyze the longitudinal virological data, we quantify the effects of IFN-α on cell-free and cell-to-cell HIV-1 infection and show that IFN-α affects TF and CC viruses differently.

## Results

### Quantification of the contribution of cell-free and cell-to-cell infection to HIV-1 spread

**Fig 1:**
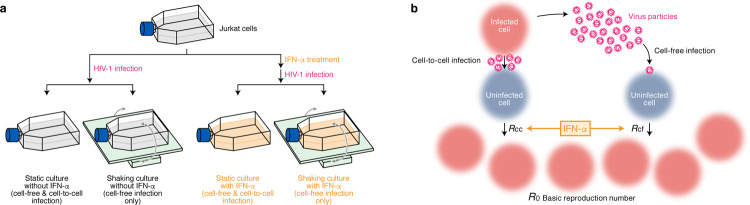
Cell culture systems and the basic reproduction number under cell-to-cell and cell-free infection with or without IFN-α. **a** An experimental scheme. Jurkat cells were infected with virus and cultured under four conditions: static or shaking cultures with or without IFN-α. IFN-α treatment (shown in orange) was performed at 24 h pre-infection. For the four cultures, the amount of viral particles in the culture supernatant and the numbers of infected and uninfected cells were routinely measured as described in Methods. The experimental results are shown in **Fig 2** (for strain NL4-3), **Fig 6** (for strain CH077_CC), and **Fig 7** (for strain CH077_TF). **b** The basic reproduction number, *R*_0_, is defined as the number of secondarily infected cells produced from an infected cell during its infectious period. In the presence of the cell-to-cell and cell-free infection, the basic reproduction number consists of two sub-reproduction numbers in which cells are infected through cell-free infection, *R*_*cf*_, and through the cell-to-cell infection, *R*_*cc*_, respectively.

A conventional cell culture system (static cell culture system) allows viruses to execute both cell-free and cell-to-cell infection. Previous studies including ours have reported that cell-to-cell infection can be blocked by gently shaking the cell culture system [19,21]. As shown in our previous study [19], gently shaking of cell culture disturbs cell-to-cell contact, but not cell-free infection (**S1 Fig**). To quantify the effect of IFN-α on cell-free and cell-to-cell infection, we adopted this experimental method (see Methods): in the static cultures of Jurkat cells, an HIV-1-susceptible human CD4^+^ T cell line [19], HIV-1 executes both cell-free and cell-to-cell infection (**Fig 1A**). On the other hand, shaking the culture of Jurkat cells allows HIV-1 to execute only cell-free infection (**Fig 1A**). Additionally, we added IFN-α in these two culture systems to analyze the effect of IFN-α on cell-free and cell-to-cell infection separately (**Fig 1B**). In these four cultures systems, we infected HIV-1 (strain NL4-3) and quantified total cell numbers, proportion of HIV-1 positive cell and the amount of virus in supernatant over time. In this study, we used 100 U/ml concentration of IFN-α. This concentration is within the range of concentrations typically used in in vitro studies [22–24], while plasma concentration is in the order of 100 pg/ml [25,26].

**Fig 2:**
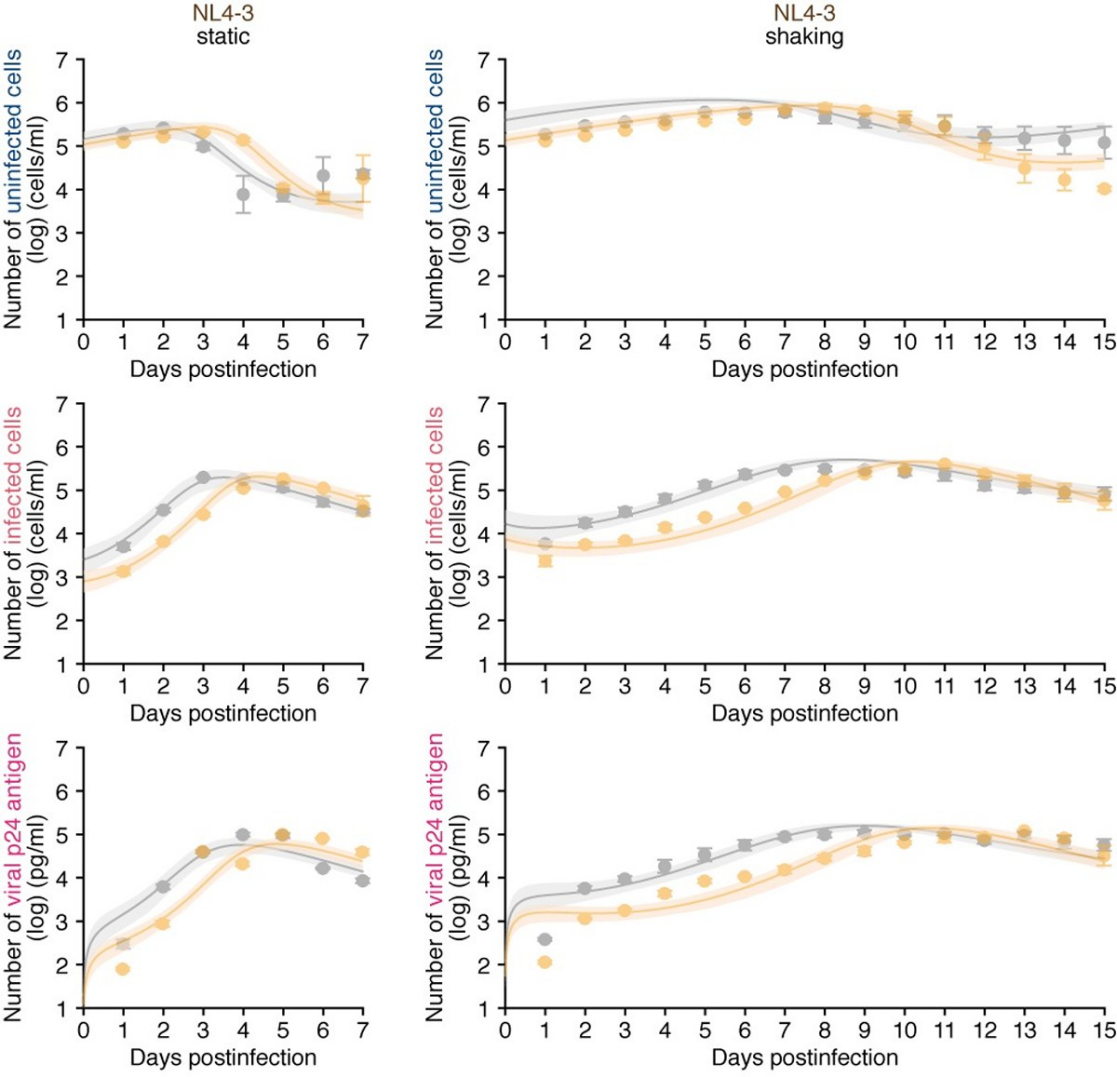
Dynamics of HIV-1 NL4-3 infection through cell-to-cell and cell-free infection without or with IFN-α. Jurkat cells were infected with HIV-1 strain NL4-3 at MOI 0.1 in the static and shaking culture under treated with IFN-α or untreated. Time-course experimental data and the fitting of Model 1 (see Methods). The time-course of experimental data for the numbers of uninfected cells (top) and infected cells (middle), and the amount of viral p24 antigen in the culture supernatant (bottom) in the static culture (left) and shaking culture (right) are shown. Gray and orange curves respectively indicate the results in the absence or presence of IFN-α treatment, respectively. The dots with error bars are the averages and SEMs of three independent experiments. The shadow regions correspond to 95% posterior predictive intervals, and the curves give the best-fit solution Model 1 to the experimental dataset.

By using the time-course data obtained from these four culture systems, we aimed to quantify the effect of IFN-α on cell-free infection and cell-to-cell infection, respectively (**Fig 1B**). As shown in **Fig 2**, HIV-1 (strain NL4-3) initially expands more rapidly in statics compared to shaking cultures. This more rapid HIV-1 expansion in static culture (**Fig 2**) strongly suggests that cell-to-cell infection are dominant compared to cell-free infection, which is consistent with our previous report [19]. As reported in our previous study [19], we applied the following model (called ’Model 0’) to quantify the effect of IFN-α from the time-course datasets:

$$\frac{dT(t)}{dt}=gT(t)(1-\frac{T(t)+I(t)}{T_{max}})-\beta T(t)V(t)-\omega T(t)I(t),$$ (1)

$$\frac{dI(t)}{dt}=\beta T(t)V(t)+\omega T(t)I(t)-\delta I(t),$$ (2)

$$\frac{dV(t)}{dt}=pI(t)-cV(t),$$ (3)

where *T*(*t*) and *I*(*t*) are the numbers of uninfected and infected cells per ml of a culture, respectively, and *V*(*t*) is the viral load measured by the amount of HIV-1 p24 per ml of culture supernatant. The parameters *g*, *T*_*max*_, *β*, *ω*, *δ*, *p* and *c* represent the maximum target cell growth rate, the maximum number of cells in the cell culture flask, the cell-free infection rate, the cell-to-cell infection rate, the death rate of infected cells, the virus production rate, and the clearance rate of virions, respectively (see **S1 Table**). Note that we fixed *ω* = 0 for the shaking cultures because the shaking inhibits the formation of cell-to-cell contacts completely in our cell culture system [21]. We also assumed that IFN-α affects only the parameters for *de novo* infection [27] (i.e., *β* and *ω* in Model 0). From cell growth experiments, *g* and *T*_*max*_ were separately estimated and fixed to be 4.75×10^−1^ d^-1^ for the static culture and 5.17×10^−1^ d^-1^ for the shaking culture for the control case, and 4.35×10^−1^ d^-1^ for the static culture and 3.98×10^−1^ d^-1^ for the shaking culture with IFN-α and *T*_*max*_ was estimated as 1.78×10^6^ (**S2 Fig and S2 Table**; see also Methods). As reported in our previous study [19], we also used *c* = 2.3 d^-1^, which is estimated from daily harvesting of viruses. The remaining common parameters, *β*, *β*_IFN_, *ω*, *ω*_IFN_, *δ* and *p*, along with the initial values for *T*(0), *I*(0) and *V*(0) in the static and the shaking cell cultures without and with IFN-α, were determined by fitting Model 0 to the data (see Methods). Note that *δ* and *p* were assumed to be common values among two conditions because IFN-α does not affect these parameters much [27,28]. In fact, even if *δ* and *p* are assumed to be different in the presence of IFN-α, we observed only a small effect on these estimated parameters. The estimated parameters of Model 0 and derived quantities are given in **S1 Table**, and the estimated initial values are summarized in **S3 Table**. The typical behavior of the model using these best-fit parameter estimates is shown together with the data in **S3 Fig**.

### Quantification of the effect of IFN-α on cell-free and cell-to-cell HIV-1 infection

**Fig 3:**
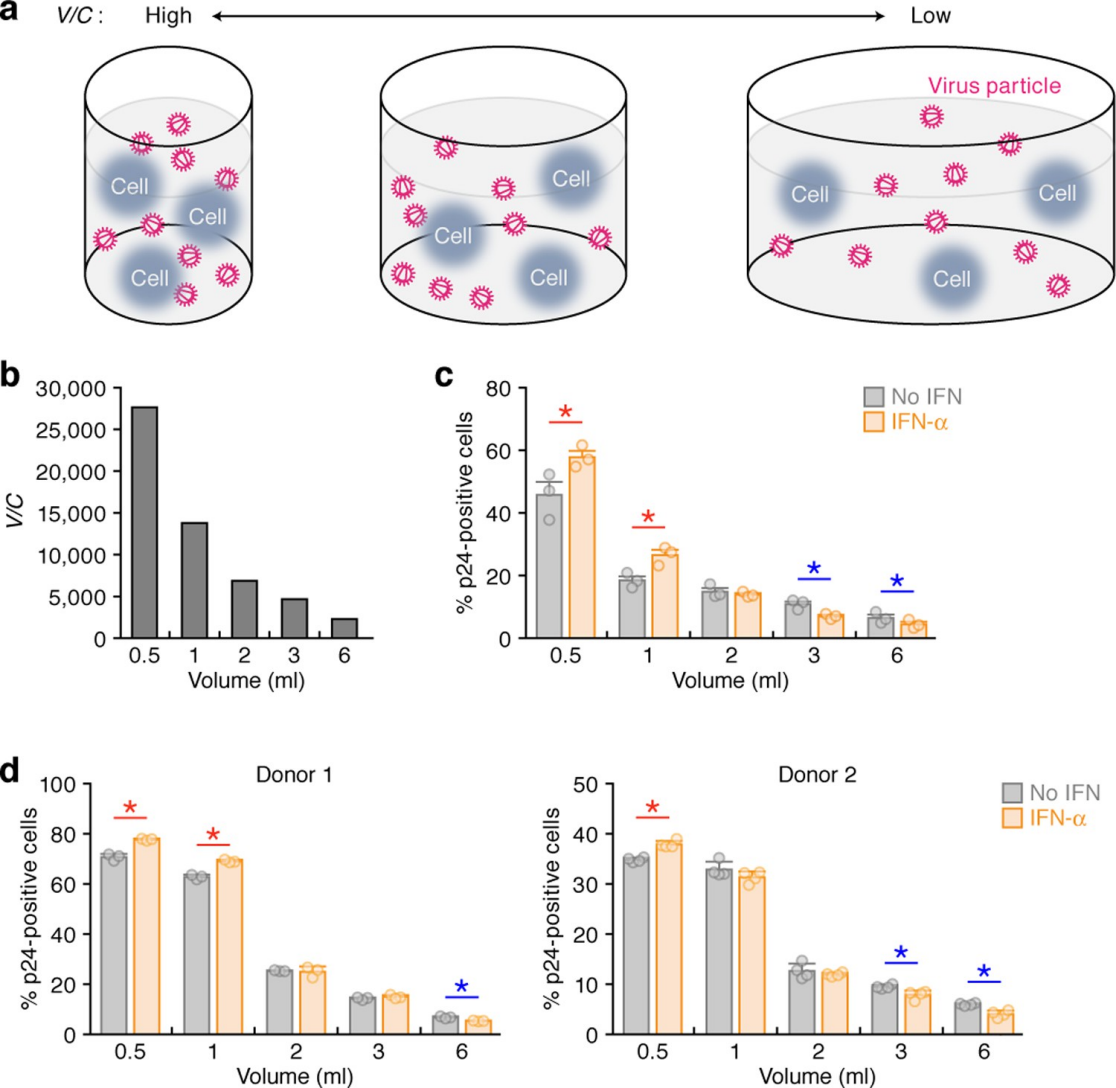
Promotion of cell-free HIV-1 infection by IFN-α under high cell-virus density. **a** A scheme of density dependence of the frequency of virus-cell contact. Nevertheless the numbers of cells and viruses are constant (cell number = 3, virus number = 9 in this panel), viruses and cells are condensed in smaller volume of culture (left panel; i.e., the value *V/C* is high), while viruses and cells are sparse in larger volume of culture (right panel; i.e., the value *V/C* is low). **b-d** Single-round infection assay under different density conditions. **b** The *V/C* value at five different culture conditions (0.5, 1, 2, 3, and 6 ml; corresponding to the experimental results shown in **Figs 3C, 7C** and **S4**) is shown. The *V/C* value is calculated as described in Methods. **c,d** Single round infection assay was performed using Jurkat-CCR5 cells (**c**) and primary human CD4^+^ T cells (**d**, 2 donors) at five different cell-virus densities with or without IFN-α. Each color circle indicates the result from one culture, and three independent experiments were performed. The horizontal bars with error bars are the averages and SEMs of three (**c,d left**) or four (**d right**) independent experiments. Asterisks indicate statistically significant differences determined by Student’s *t* test (*p*<0.05).

To quantify the spread of the virus by the two infection modes, cell-free and cell-to-cell infection, we derived and calculated the basic reproduction number *R*_0_ = *R*_*cf*_+*R*_*cc*_ (see **S1 Table**) [19]. Here *R*_*cf*_ and *R*_*cc*_ correspond to the average number of newly infected cells produced from one infected cell through the cell-free and cell-to-cell infection, respectively. When we consider the effect of IFN-α on *R*_*cf*_ (i.e., cell-free infection) and *R*_*cc*_ (i.e., cell-to-cell infection) (**S3B–S3E Fig**), IFN-α treatment decreased *R*_*cc*_ by 16% (**S1 Table** and **S3C Fig**). However, surprisingly, IFN-α treatment increased *R*_*cf*_ by 19% (**S1 Table** and **S3D Fig**) with statistical significance (*p* = 0.000122 by Brunner Munzel test). These findings raise the possibility that IFN-α can enhance cell-free HIV-1 infection in certain conditions. Since the number of cells and viral particles in the culture increase during the experiments (**Fig 2**), we asked whether the higher density of viruses and cells influence the effect of IFN-α on HIV-1 infection. To assess this hypothesis, we performed experiments with different densities of viruses and cells where the fraction of infected target cells was assessed at 48 hr post-infection, a time at which we expect there was only a single-round of infection. Here we defined the density of viruses and cells as *V/C* (the ratio of the number of viral particles to the number of cells in a culture divided by the culture volume) (**Fig 3A**; see Methods for detail). The *V/C* values under different conditions (0.5 to 6 ml culture media) are summarized in **Fig 3B**. When V/C is small, i.e. when the volume is 3 or 6 ml, there is a lower percentage of infected cells in the presence of IFN-α compared to when it is absent (**Fig 3C**; indicated with blue asterisks). This result is consistent with previous reports [22,29]. Also, while this change is statistically significant, p< 0.05, the effect size is small and may not be biologically relevant. In sharp contrast, IFN-α enhanced HIV-1 infection when *V/C* is high (**Fig 3C**; indicated with red asterisks). When the *V/C* values increased, the density-dependent promotion effect on HIV-1 infection by IFN-α also increased (**Fig 3C**) presumably because of the increase of the contact frequency of viral particles and cells. Moreover, the effect of IFN-α in promoting HIV-1 infection is observed not only in Jurkat cells (**Fig 3C**) but also in SupT11 cells, another human CD4^+^ T cell line (**S4 Fig**), suggesting that this enhancing effect is not specific for this cell line. Additionally, the density-dependent effect of IFN-α in promoting HIV-1 infection was observed in primary human CD4^+^ T cells from two different donors (**Fig 3D**). Therefore, these results suggest that IFN-α modest promotes cell-free HIV-1 infection when *V/C* is high.

**Fig 4:**
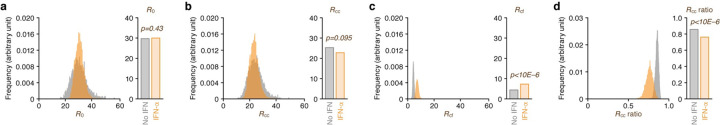
Distributions of basic reproduction numbers of HIV-1 NL4-3 infection. **a-d** The comparison of basic reproduction numbers in the absence or presence of IFN-α. (Left) The distributions of *R*_0_ (**a**), *R*_*cc*_ (**b**), and *R*_*cf*_ (**c**), computed from the all accepted parameters by the MCMC methods, are shown. The contribution of the cell-to-cell infection (i.e. *R*_*cc*_/(*R*_*cc*_+*R*_*cf*_)) is shown in panel **d**. In these analyses, we sampled 20,000 parameter sets from MCMC computation among 150,000 samples. For detail, see Methods. (Right) The bars indicate the mean values computed by the MCMC method. Orange and gray respectively indicate the data with and without IFN-α treatment. The *p* values are calculated by Brunner Munzel test.

Since the experiments (**Fig 3**) suggested the facilitating effect of IFN-α on cell-free HIV-1 infection depends on *V/C*, we expanded Model 0 to include the effect of IFN-α depending on the density of viruses and cells. A simple modification to consider this effect is assuming a virus density-dependent cell-free infection rate as follows:

$$\frac{dT(t)}{dt}=gT(t)\left(1-\frac{T(t)+I(t)}{K}\right)-\beta \left(1+k\frac{V(t)}{V_{50}+V(t)}\right)T(t)V(t)-\omega T(t)I(t),$$ (4)

$$\frac{dI(t)}{dt}=\beta \left(1+k\frac{V(t)}{V_{50}+V(t)}\right)T(t)V(t)+\omega T(t)I(t)-\delta I(t),$$ (5)

$$\frac{dV(t)}{dt}=pI(t)-cV(t),$$ (6)

where *k* and *V*_50_ describe the effect of IFN-α in promoting cell-free infection by IFN-α, with *k* being the maximum effect and *V*_50_ being the viral load inducing 50% of the maximum promotive effect. Note that in the absence of IFN-α, we set *k* = 0, which corresponds to Model 0. Our expanded model (called ’Model 1’) using the best-fit parameter estimates and initial values in **Tables 1** and S4 is shown together with the data in **Fig 2**, which reveals that Model 1 also describes these *in vitro* data. In similar manner, we calculated the distributions of *R*_0_, *R*_*cf*_, and *R*_*cc*_, respectively (**Fig 4A–4D**). *R*_*cc*_ was decreased to 90% by IFN-α, which difference approached significance (**Fig 4A**; *p* = 0.095). Consistent with other virological experiments (**Fig 3**), IFN-α treatment significantly increased *R*_*cf*_, here by 66% (**Fig 4C**; *p*<1×10^−6^), although the contribution of *R*_*cf*_ to *R*_0_ is relatively minor (**Fig 4D**; *p*<1×10^−6^). Due to the balance of decreased *R*_*cc*_ and increased *R*_*cf*_ IFN-α, there was no statistical difference in *R*_0_ (**Fig 4A**; *p* = 0.43). These findings suggest that IFN-α suppresses HIV-1 spread by suppressing cell-to-cell infection rather than the promotive effect for cell-free infection.

**Table 1:** Estimated parameters fitting the experimental data of HIV-1 strain NL4-3 by Model 1.

Parameter name	Symbol	Unit	Without IFN-α		With IFN-α	
			Mean	95% CI*	Mean	95% CI*
Rate constant for cell-free infection	*β*	10^−6^×(p24 day)-1	5.117	4.164–6.286	8.590	6.070–11.96
Rate constant for cell-to-cell infection	*Ω*	10^−6^×(cell day)^-1^	9.614	6.615–13.28	8.716	6.301–11.74
Death rate of infected cells	*δ*	day^-1^	0.6712	0.6343–0.7093	0.6712	0.6343–0.7093
Production rate of total viral protein	*p*	day^-1^	0.7304	0.6519–0.8131	0.7304	0.6519–0.8131
The parameter in hill function	*v*_50_	10^6^×p24	---	---	6.632	3.997–10.45
The promotion effect of virus density per cell	*k*	10^−4^	---	---	7.166	4.224–11.34
Basic reproduction number through cell-to-cell infection	*R*_*cc*_ ($=\frac{\omega K}{\delta }$)	-	25.35	16.87–36.03	22.84	17.36–29.57
Basic reproduction number through cell-free infection	*R*_*cf*_ ($=\frac{p\beta K}{c\delta }$)	-	4.273	3.365–5.359	7.104	5.628–8.932
Basic reproduction number	*R*_0_ (= *R*_*cc*_+*R*_*cf*_)	-	29.63	20.52–40.99	29.94	24.78–36.18
Contribution of cell-to-cell infection	$\frac{R_{cc}}{R_{cc}+R_{cf}}$	-	0.8533	0.8130–0.8866	0.7602	0.6801–0.8293

*CI: credible interval.

### Effect of IFN-α treatment on the cellular transcriptome

Next, we addressed the molecular factors that are involved in the suppression of cell-to-cell HIV-1 infection by IFN-α. It is well known that IFN-α induces the expression of ISGs and certain ISGs restrict viral replication (reviewed in references [30,31]). To assess the ISGs up-regulated in IFN-α-treated Jurkat cells, we performed RNA-sequencing (RNA-Seq) analysis. As shown in **S5A Fig**, the dimensionality reduction by t-distributed stochastic neighbor embedding (tSNE) visualized that IFN-α treatment drastically changes the cellular transcriptome, while the cell culture condition (i.e., static or shaking) did not affect the cellular gene expression pattern (the lists of differentially expressed genes [DEGs] and their ontology are shown in **S5–S7 Tables**). Particularly, some ISGs already known to restrict HIV-1 replication such as *MX2* [32,33], *IFITM1-3* [34,35], *BST2* [36,37], *TRIM56* [38], and *N4BP1* [39], are significantly up-regulated in IFN-α-treated Jurkat cells (**S5B Fig**).

### Effect of IFN-α treatment on CC and TF viruses

**Fig 5:**
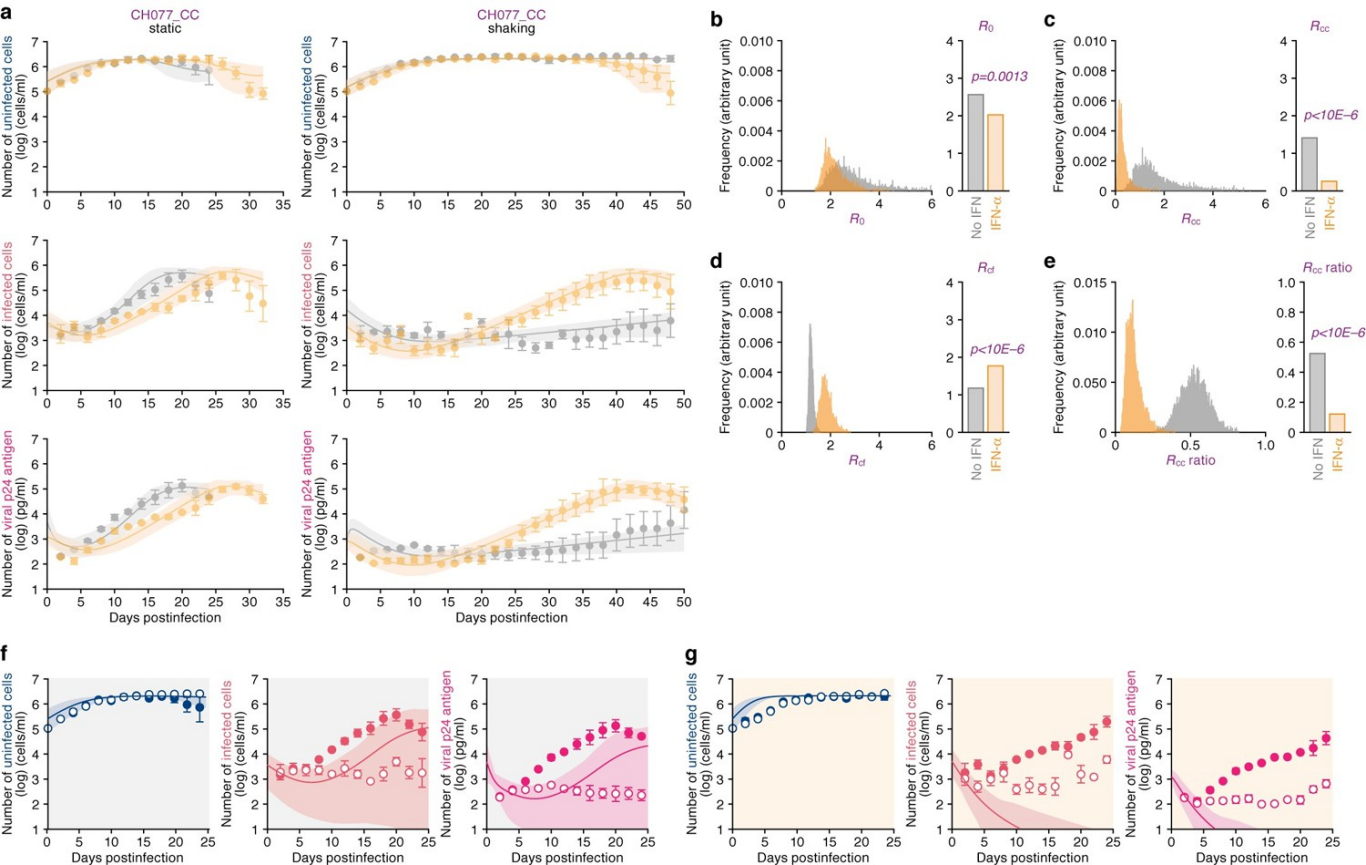
Dynamics of HIV-1 CH077_CC infection in static and shaking cultures without or with IFN-α. Jurkat-CCR5 cells were infected with HIV-1 strain CH077_CC at MOI 0.05 in the static and shaking cultures under treated with IFN-α or untreated. **a** Longitudinal experimental data and the fitting of Model 1 to the data (see Methods). The time-course of experimental data for the numbers of uninfected cells (top) and infected cells (middle), and the amount of viral p24 antigen in the culture supernatant (bottom) in the static culture (left) and shaking culture (right) are shown. Gray and orange curves respectively indicate the results in the absence or presence of IFN-α treatment. The dots with error bars are the averages and SEMs of three independent experiments. The shadow regions correspond to 95% posterior predictive intervals, and the curves give the best-fit solution Model 1 to the experimental dataset. **b-e** The comparison of basic reproduction numbers in the absence or presence of IFN-α. (Left) The distributions of *R*_0_ (**b**), *R*_*cf*_ (**c**), and *R*_*cc*_ (**d**), computed from all the accepted parameters are shown. The contribution of cell-to-cell infection (i.e. *R*_*cc*_/(*R*_*cc*_+*R*_*cf*_)) is shown in panel **e**. (Right) The bars indicate the mean values computed by the MCMC method. Orange and gray respectively indicate the data with and without IFN-α treatment. The *p* values are calculated by the Brunner Munzel test. **f,g** Virtual simulation of cell-to-cell infection of HIV-1 strain CH077_CC. Using our estimated parameters (**Table 2**), pure cell-to-cell infection is simulated *in silico* (solid curves). The simulated values are located between the time course of the experimental data under the static conditions (closed circles) where both cell-free and cell-to-cell infection can occur and those under the shaking conditions (open circles) where only cell-free infection can occur. The shadowed regions correspond to 95% posterior predictive intervals. Panels indicate the results in the absence (**f**) or presence (**g**) of IFN-α treatment, respectively.

Although the opposing effects of IFN-α, inhibition of cell-to-cell infection and promotion of cell-free infection, were observed (**Figs 2** and **4**), a laboratory strain (NL4-3) was used in these experiments. As described in the Introduction, clinical isolates are classified into TF and CC viruses based on the clinical stage of the infected individuals from which they were isolated, and the phenotype of these viruses, particularly with regard to the sensitivity to IFN-α is clearly different: TF viruses are resistant to the antiviral effect of IFN-α, while CC viruses are relatively sensitive [5,6,8]. To investigate the effect of IFN-α on cell-free and cell-to-cell infection of TF and CC viruses, we used clinical strains isolated from a patient CH077 [3,40]; strain CH077_TF is the infectious molecular clone (IMC) of the TF virus from this patient, while strain CH077_CC is the IMC isolated from this patient during the chronic phase of infection (after 6 months postinfection) [6]. We first performed experiments using CH077_CC with or without IFN-α under static and shaking conditions, and Model 1 was fit to the experimental data (**Fig 5A**). Here we note that Model 0 does not fit the experimental data well (not shown). The estimated parameters and initial values for Model 1 are listed in **Tables 2** and **S8**, and the computed basic reproduction numbers are shown in **Fig 5B–5E**. Similar to the results from HIV-1 strain NL4-3 (**Fig 4**), IFN-α decreased the *R*_0_ of CH077_CC (**Fig 5B**; *p* = 0.0013). In particular, *R*_*cc*_ of CH077_CC was severely decreased by IFN-α treatment (**Fig 5C**; 82% reduction, *p*<1×10^−6^), suggesting that the vulnerability of CC viruses to IFN-α is attributed to the sensitivity to IFN-α’s antiviral effect on cell-to-cell infection. Moreover, IFN-α reduced *R*_*cc*_ to less than 1 (**Fig 5C**). On the other hand, we also found that *R*_*cf*_ of CH077_CC is significantly increased by IFN-α treatment (**Fig 5D**; 50% increase, *p*<1×10^−6^) to a value close to 2, which when combined with *R*_*cc*_ being less than 1 implies that cell-free infection in the main route of CH077_CC infection in the presence of IFN-α. Surprisingly, IFN-α significantly reduces the ratio of *R*_*cc*_ to *R*_0_ of CH077_CC (**Fig 5E**; to less than 50%).

**Table 2:** Estimated parameters fitting the experimental data of HIV-1 strain CH077_CC by Model 1.

Parameter name	Symbol	Unit	Without IFN-α		With IFN-α	
			Mean	95% CI*	Mean	95% CI*
Rate constant for cell-free infection for control case	*β*	10^−6^×(p24 day)^-1^	1.301	0.7629–2.261	1.910	1.213–3.121
Rate constant for cell-to-cell infection for control case	*ω*	10^−6^×(cell day)^-1^	0.3215	0.1958–0.4990	0.07196	0.03631–0.1283
Death rate of infected cells	*δ*	day^-1^	0.5148	0.3049–0.8034	0.5148	0.3049–0.8034
Production rate of total viral protein	*p*	day^-1^	0.5358	0.3852–0.7447	0.5358	0.3852–0.7447
The parameter in hill function	*v*_50_	10^7^×p24	-	-	2.626	1.670–15.70
The effect of cell density per virus	*k*	-	-	-	59.50	12.31–161.1
Basic reproduction number through cell-to-cell infection	*R*_*cc*_ ($=\frac{\omega K}{\delta }$)	-	1.382	0.6327–2.721	0.2546	0.08531–0.6396
Basic reproduction number through cell-free infection	*R*_*cf*_ ($=\frac{p\beta K}{c\delta }$)	-	1.171	1.056–1.334	1.757	1.445–2.188
Basic reproduction number	*R*_0_ (= *R*_*cc*_+*R*_*cf*_)	-	2.553	1.752–3.945	2.012	1.544–2.797
Contribution of cell-to-cell infection	$\frac{R_{cc}}{R_{cc}+R_{cf}}$	-	0.5225	0.3559–0.6949	0.1197	0.05450–0.2341

*CI: credible interval.

Next, we studied the effect of eliminating the contribution of cell-free infection *in silico* (i.e., we fixed *β* = 0) to elucidate how the total blocking of cell-free infection by IFN-α treatment could affect the time course of infection. We thus simulated the ’pure’ time-course of cell-to-cell infection dynamics using the last 100,000 among 150,000 accepted Markov Chain Monte Carlo (MCMC) parameter sets (solid curve in **Fig 5F** and **5G**). Since CH077_CC infection in the presence of IFN-α cannot be maintained by only cell-to-cell infection the number of infected cells and virions are predicted to approach 0 (**Fig 5G**), cell-free infection is the dominant model of CH077_CC transmission during the entire course of infection (**Fig 5F**). Altogether, these findings suggest that IFN-α is detrimental for cell-to-cell HIV-1 CH077_CC infection, while it is beneficial for cell-free infection.

**Fig 6:**
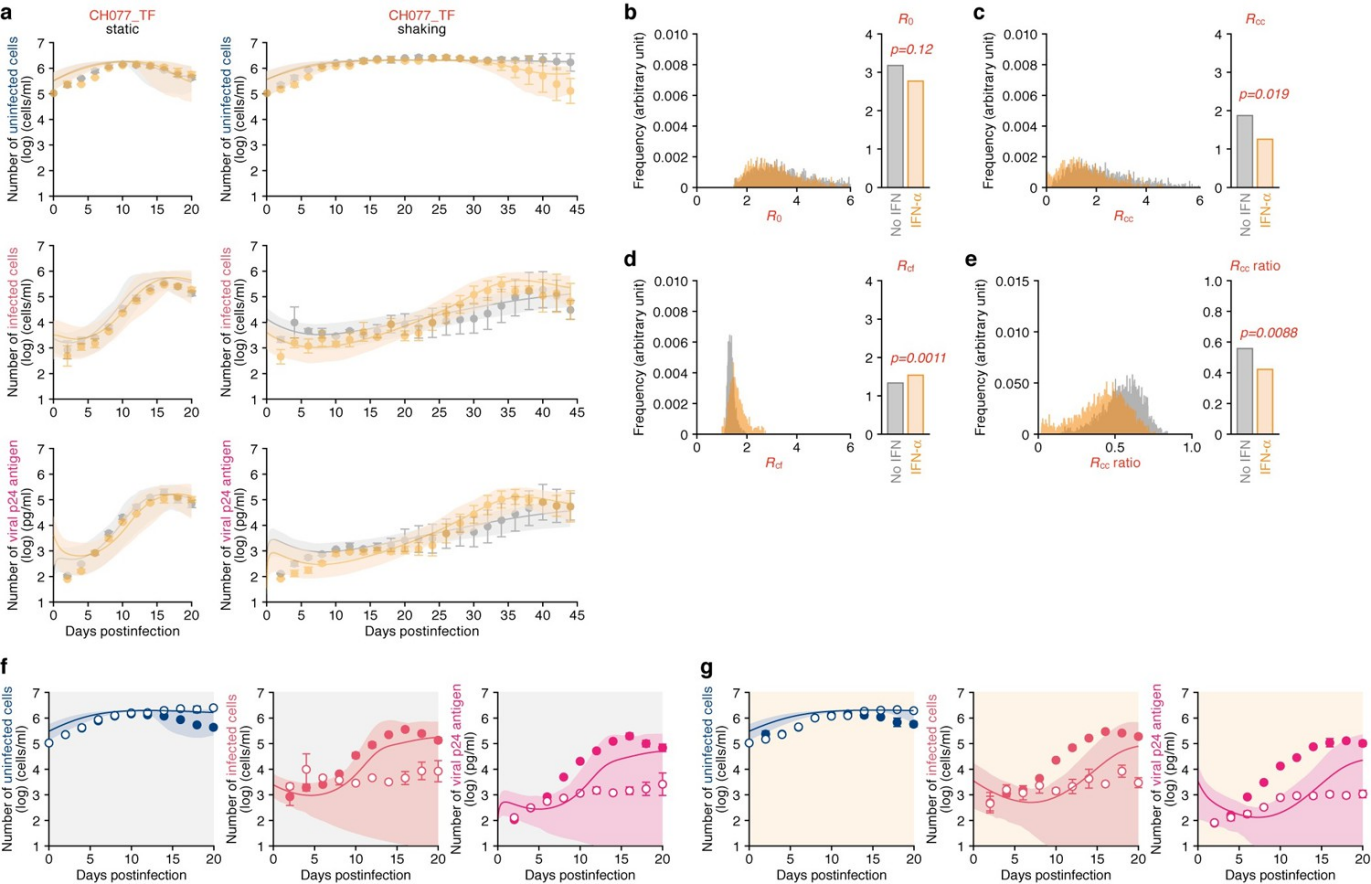
Dynamics of HIV-1 CH077_TF infection in static and shaking cultures without or with IFN-α. Jurkat-CCR5 cells were infected with HIV-1 strain CH077_TF at MOI 0.05 in the static and shaking culture under treated with IFN-α or untreated. **a** Longitudinal experimental data and the fitting of Model 1 to the data (see Methods). The time-course of experimental data for the numbers of uninfected cells (top) and infected cells (middle), and the amount of viral p24 antigen in the culture supernatant (bottom) in the static culture (left) and shaking culture (right) are shown. Gray and orange curves respectively indicate the results in the absence or presence of IFN-α treatment. The dots with error bars are the averages and SEMs of three independent experiments. The shadow regions correspond to 95% posterior predictive intervals, and the curves give the best-fit solution Model 1 to the experimental dataset. **b-e** The comparison of basic reproduction numbers in the absence or presence of IFN-α. (Left) The distributions of *R*_0_ (**b**), *R*_*cf*_ (**c**), and *R*_*cc*_ (**d**), computed from all the accepted parameters are shown. The contribution of cell-to-cell infection (i.e. *R*_*cc*_/(*R*_*cc*_+*R*_*cf*_)) is shown in panel **e**. (Right) The bars indicate the mean values computed by MCMC methods. Orange and gray respectively indicate the data with and without IFN-α treatment. The *p* values are calculated by the Brunner Munzel test. **f,g** Virtual simulation of cell-to-cell infection of HIV-1 strain CH077_ TF. Using our estimated parameters (**Table 3**), the pure cell-to-cell infection is simulated *in silico* (solid curves). The simulated values are located between the time course of experimental data under the static conditions (closed circles) where both cell-free and cell-to-cell infection can occur and those under the shaking conditions (open circles) where only cell-free infection can occur. The shadowed regions correspond to 95% posterior predictive intervals. Panels indicate the results in the absence (**f**) or presence (**g**) of IFN-α treatment, respectively.

We next performed similar experiments using CH077_TF (**Fig 6A and Tables 3** and **S9**). Similar to strain CH077_CC (**Fig 5D**), IFN-α increased *R*_*cf*_ of CH077_TF (**Fig 6D**; *p* = 0.0011). These results suggest that the effect of IFN-α on promoting cell-free HIV-1 infection is broadly applicable, although its extent is different among strains. Additionally, it was noteworthy that *R*_*cc*_ of CH077_TF was slightly decreased by IFN-α treatment (**Fig 6C**; 34% reduction), and the reduction level was clearly less than that of CH077_CC (**Fig 5C**; 82% reduction). Moreover, although the *R*_*cc*_ ratio of CH077_CC was severely decreased by IFN-α treatment (**Fig 5E**), that of CH077_TF was relatively less changed (**Fig 6E**). In total, the *R*_0_ of CH077_TF was not significantly decreased by IFN-α treatment (**Fig 6B**; *p* = 0.12). Compared to CH077_CC (**Fig 5F** and **5G**), IFN-α did not critically affect the kinetics of cell-to-cell infection of CH077_TF (**Fig 6F** and **6G**). Altogether, these findings suggest that CH077_TF is resistant to any IFN-α-mediated antiviral effect, which is attributed here to the tolerance to the inhibition of cell-to-cell infection by IFN-α.

**Table 3:** Estimated parameters fitting the experimental data of HIV-1 strain CH077_TF by Model 1.

Parameter name	Symbol	Unit	Without IFN-α		With IFN-α	
			Mean	95% CI*	Mean	95% CI*
Rate constant for cell-free infection	*β*	10^−6^×(p24 day)^-1^	1.237	0.4980–2.546	1.437	0.5944–2.816
Rate constant for cell-to-cell infection	*ω*	10^−6^×(cell day)^-1^	0.4664	0.1847–0.8830	0.3215	0.04808–0.6825
Death rate of infected cells	*δ*	day^-1^	0.5317	0.2201–1.044	0.5317	0.2201–1.044
Production rate of total viral protein	*p*	day^-1^	0.7271	0.3245–1.411	0.7271	0.3245–1.411
The parameter in hill function	*v*_50_	10^4^×p24	-	-	8.401	0.8169–38.45
The effect of cell density per virus	*k*	-	-	-	1.402	0.3179–4.199
Basic reproduction number through cell-to-cell infection	*R*_*cc*_ ($=\frac{\omega K}{\delta }$)	-	1.879	0.5719–4.144	1.238	0.1199–2.753
Basic reproduction number through cell-free infection	*R*_*cf*_ ($=\frac{p\beta K}{c\delta }$)	-	1.321	1.160–1.552	1.532	1.184–2.085
Basic reproduction number	*R*_0_ (= *R*_*cc*_+*R*_*cf*_)	-	3.201	1.826–5.563	2.770	1.733–4.344
Contribution of cell-to-cell infection	$\frac{R_{cc}}{R_{cc}+R_{cf}}$	-	0.5569	0.3088–0.7441	0.4190	0.05897–0.6607

*CI: credible interval.

### Contribution of LY6E, an ISG, to the promotion of cell-free HIV-1 infection

**Fig 7:**
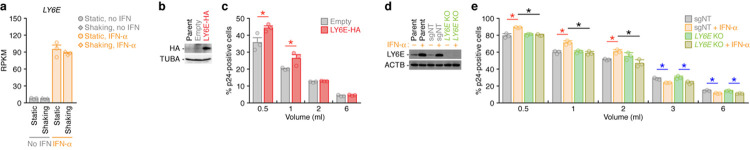
Contribution of LY6E to the enhancement of cell-free HIV-1 infection. **a** The expression levels of *LY6E*. Each symbol indicates the result from one replicate, and the bars with error bars indicate the averages with SEMs of three independent replicates. RPKM, reads per kilobase of exon per million mapped reads. **b,c** Effect of LY6E overexpression on cell-free HIV-1 infection. **b** Western blotting. Parental Jurkat-CCR5 cells (’Parent’), empty vector-transduced cells (Empty), and LY6E-HA-transduced cells (’LY6E-HA’) were prepared as described in Methods, and the LY6E-HA expression is detected by Western blotting. A representative result is shown. Alpha-tubulin (TUBA) is used as an internal control. **c** Single-round infection assay under different density conditions. The *V/C* at different five culture conditions (0.5, 1, 2, and 6 ml) corresponds to that shown in **Fig 3A**. Single round infection assay was performed at four different cell-virus densities. **d,e** Effect of *LY6E* KO on cell-free HIV-1 infection. **d** Western blotting. Parental Jurkat-CCR5 cells (’Parent’), non-target sgRNA-transduced cells (’SgNT’), and *LY6E* KO cells (*’LY6E* KO’) were prepared as described in Materials and Methods, and endogenous LY6E expression is detected by Western blotting. A representative result is shown. TUBA is used as an internal control. **e** Single-round infection assay under different density conditions. The *V/C* at different five culture conditions (0.5, 1, 2, 3 and 6 ml) corresponds to that shown in **Fig 3A**. Single round infection assay was performed at five different cell-virus densities. In panels **c** and **e**, each dot indicates the result from one culture, and three independent experiments were performed. The bars with error bars are the averages and SEMs of three independent experiments. Asterisks indicate statistically significant differences determined by Student’s *t* test (*p*<0.05).

As described above, the promotion of cell-free HIV-1 infection by IFN-α treatment was broadly observed in multiple strains (**Figs 4C, 5D** and **6D**). These observations raise the possibility that certain ISG(s) positively regulate cell-free HIV-1 infection. Regarding this issue, it has been reported that an ISG, LY6E, promotes infection by various viruses such as flaviviruses (e.g., West Nile virus, yellow fever virus, dengue virus, and Zika virus), influenza A virus, and HIV-1 [41–45]. In fact, our RNA-Seq data indicated the up-regulation of *LY6E* in IFN-α-treated cells (**Fig 7A**). To assess whether LY6E can contribute to the enhancement of cell-free HIV-1 infection, we prepared a Jurkat cell line transduced with HA-tagged LY6E (Jurkat/LY6E-HA). The expression of LY6E-HA was detected by Western blotting (**Fig 7B**), and the expression levels of CD4 and CCR5 were not affected by LY6E-HA expression (**S6 Fig**). By using the Jurkat/LY6E-HA cells and control cells (empty vector-transduced cells), we performed single-round infection experiments using different densities of viruses and cells (**Fig 3A** and **3B**). As shown in **Fig 7C**, cell-free HIV-1 infection was promoted by LY6E-HA expression when *V/C* is high. Moreover, to investigate the effect of endogenous LY6E on HIV-1 infection, we prepared *LY6E* knock-out (KO) Jurkat-CCR5 cells using the CRISPR/Cas9 system. The KO process did not affect the expression levels of CD4 and CCR5 (**S7 Fig**). Western blotting verified that the expression of endogenous LY6E was upregulated by IFN-α treatment in parental and non-target guide RNA-transduced cells but not in *LY6E* KO cells (**Fig 7D**). Finally, the single-round infection experiments using different densities of viruses and cells showed that the enhancement of HIV-1 infection by IFN-α treatment at a high density is abolished by *LY6E* KO (**Fig 7E**). Altogether, these results strongly suggest that LY6E contributes to the promotion of cell-free HIV-1 infection by IFN-α.

## Discussion

**Fig 8:**
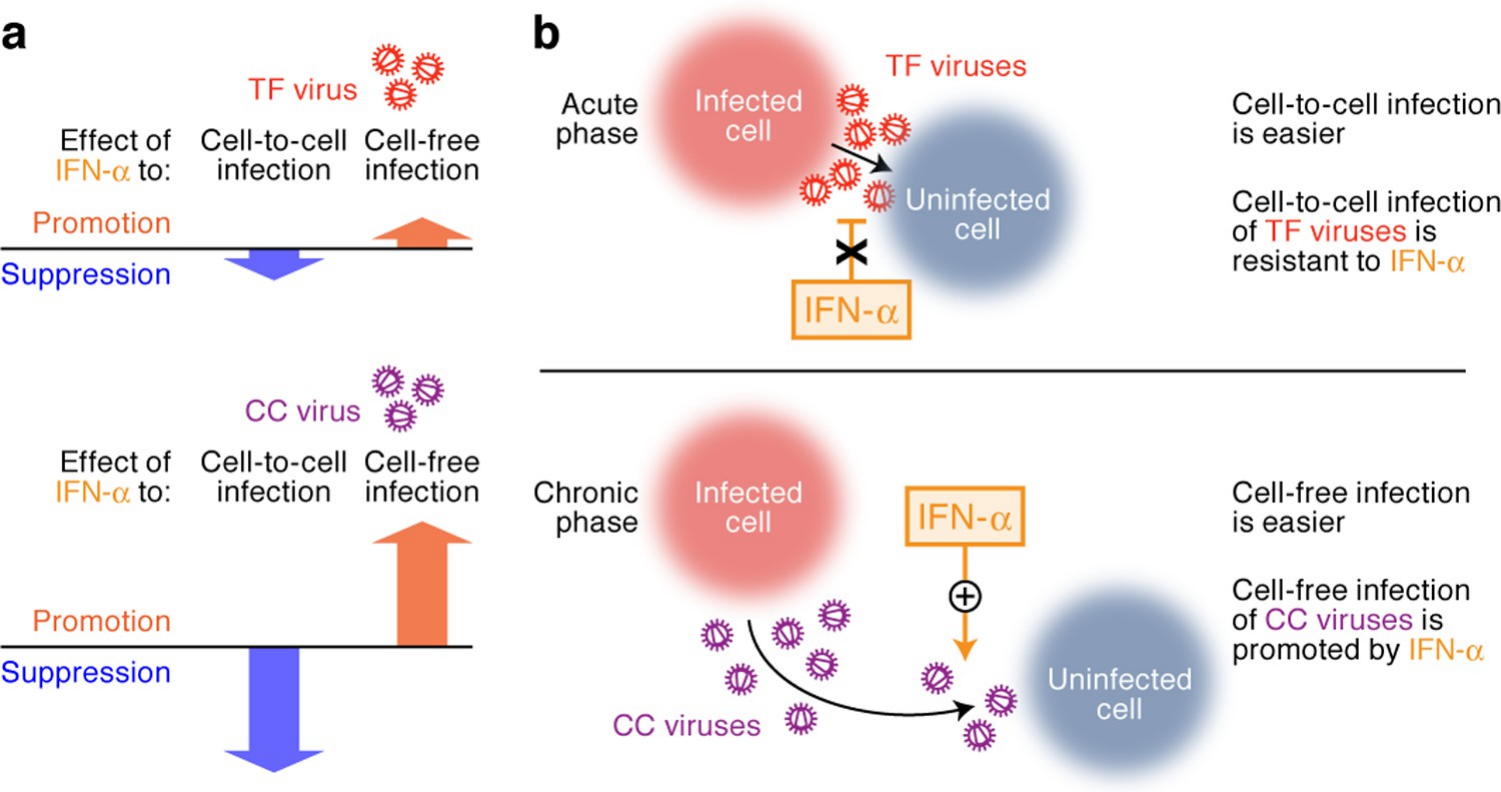
Graphical summary of this study. **a** Summary of phenotypic properties of TF and CC viruses observed in this study. CC virus (bottom) is positively (for cell-free infection) and negatively (cell-to-cell infection) sensitive to IFN-α stimulation, while TF viruses (top) is less affected by IFN-α stimulation. **b** Proposed hypothesis raised by this study. The detailed explanation is described in Discussion.

Through a combined experimental-mathematical approach, we quantitatively distinguished the contributions of cell-free (parameter *R*_*cf*_) and cell-to-cell (parameter *R*_*cc*_) infection in multi-round HIV-1 replication in cell culture and evaluated the effect of IFN-α treatment on these two viral infection modes. By using two clinical HIV-1 isolates from the same HIV-1 infected patient, one of which was a TF virus and other a CC virus, we demonstrated that TF virus is relatively resistant to IFN-α’s antiviral effect, while CC virus is relatively sensitive (**Fig 8A**). In particular, our experimental-mathematical investigation revealed that the cell-to-cell infection of TF virus is relatively resistant to the antiviral effect of IFN-α, while the cell-to-cell infection of CC virus is strongly suppressed by IFN-α. Intriguingly, we demonstrated that IFN-α promotes cell-free HIV-1 infection, and particularly, CC virus benefits from this enhancing effect. To our knowledge, this is the first investigation addressing the phenotypic difference in the replication kinetics of a pair of TF and CC viruses in the presence of IFN-α through a joint experimental-mathematical approach.

Previous studies using mathematical models to examine the effects of IFN on HIV infection have discussed the impact of IFN on de novo infections and virus production based on experimental data from Jurkat cells infected with the NL4-3 strain [46]. Our analysis does not take into account the concentration-dependent effects of IFN and its impact on virus production. In addition, the values of basic reproduction numbers, *R*_0_, estimated in this study were higher (NL4-3) and lower (TF and CC) than those obtained from in vivo analysis of subtype B strains in previous study [47]. The estimates may be different for other subtypes (e.g. subtype C). Since the conditions in in vitro cell culture are regarded as fundamentally different from the environment of immune cells in vivo, a direct comparison of in vivo and in vitro R0 estimates would not be of great significance. The point to emphasize here is that our result does provide useful insights in terms of comparing the differences between clinical isolates of TF and CC in terms of IFN impact on the mode of infection.

One possible limitation of our mathematical model is that it does not take into account the delay in virus production due to the replication cycle of the virus in the cell. It has been reported that the delay in virus production affects the estimate of the basic reproduction number, *R*_0_ [47]. The effect of such a delay in the dynamics of viral infection in the cell remains an issue to be explored in the future. In addition, our estimates of rate constants for infections, *β* and *ω*, may be the average of time-varying infection rate due to ignored effects such as prevention of infection by endogenous IFNs produced by infected cells. Another limitation in our study is that the time of IFN addition was not considered. The time for IFN pre-treatment is fixed in our experiment, while time of IFN addition is important parameter for determining IFN effect on virus infection in many studies [48–51]. Further consideration will be needed to investigate this time of addition dependence in IFN effects.

Here we described a new aspect of IFN-α’s effect on HIV-1 infection: IFN-α potently enhances cell-free infection when the parameter *V/C* is high. We also revealed that an ISG, LY6E, is up-regulated by IFN-α treatment and promotes HIV-1 infection. Although the other key ISGs might be responsible for this effect, in fact, it has been recently reported that LY6E enhances infection of HIV-1 [42] and various viruses such as yellow fever virus, dengue virus, Zika virus and influenza A virus [41] by promoting the viral entry step. Since this promoting effect is observed in multiple viral strains (NL4-3, CC_CH077 and TF_CH077) and multiple cell lines (Jurkat and SupT11 cells) as well as primary human CD4^+^ T cells, the promotion of HIV-1 infection by IFN-α is not specific for certain viral strains or CD4^+^ T cell lines. Moreover, *LY6E* is up-regulated in the CD4^+^ T cells of HIV-1-infected individuals during the chronic phase of infection [52–55]. Therefore, our findings suggest that LY6E may play a promoting role in HIV-1 replication in patients during the chronic phase of infection.

As described above, consistent with our findings, the promotion of HIV-1 infection was observed in a previous report [42]. However, the same group recently reported that LY6E overexpression suppresses HIV-1 (strain NL4-3) infection in Jurkat cells but enhances it in SupT1 cells [56]. The difference between this previous report [56] and our findings might be due to differences in the experimental setup, but the precise reason(s) remain unclear. Further, a recent study showed that LY6E suppresses coronavirus infection by impairing viral fusion [57], which is opposite to the observations in some other RNA viruses (e.g., yellow fever virus and dengue virus) [41]. Altogether, the actions of LY6E on virus infection seem to be tricky, and therefore, further investigation will be needed to fully elucidate the pro- or anti-viral roles of LY6E in virus infections and replications.

In spite of the up-regulation of antiviral ISGs such as *IFITM1*, *IFITM3* and *MX2* by IFN-α treatment, why was the IFN-α-mediated antiviral effect canceled in the following two experiments: (1) multi-round virus infection in the shaking culture that only allows cell-free infection; and (2) single-round virus infection at relatively higher densities of cells and viruses? These are reminiscent of the saturation of the antiviral effects mediated by anti-HIV-1 drugs [16,58–60]. When the parameter *V/C* is high, the antiviral effect by the drugs that target the early steps of HIV-1 replication (e.g., entry and reverse transcription) is saturated because of the higher amount of input virus per cell (reviewed in reference [15]). Such a saturation of antiviral effect is also observed in the case of the antiviral restriction factors that block the early step of viral replication. For instance, murine Fv1 [61,62] and TRIM5α [63,64] impair the post-entry step of viral replication. However, when the amount of input virus is high (i.e., the parameter *V/C* is high), the antiviral effects mediated by murine Fv1 [65] and TRIM5α [65,66] are saturated and canceled (reviewed in reference [67]). Similarly, two well-studied antiviral ISGs, IFITMs [34,35,68–70] and MX2 [32,33] respectively restrict the entry and post-entry steps of HIV-1 replication. Therefore, when the parameter V/C is high, it might be reasonable to consider that the antiviral effect by IFN-α treatment is saturated, while only the promoting effect on virus infection by IFN-α is maintained. In fact, the antiviral effect of IFN-α in some CD4^+^ T cell lines including Jurkat, SupT1, CEM-SS cells can be saturated dependently on the amount of input virus, although IFN-α exhibits a robust antiviral effect in myeloid cell lines such as THP-1 and U937 cells and monocyte-derived macrophages regardless of the amount of input virus [22,71]. Therefore, in CD4+ T cells, our findings suggest that IFN-α may not strongly induce an antiviral effect, but rather, promotes cell-free HIV-1 infection when V/C is high. On the other hand, in the case of cell-to-cell spread, the ability of LY6E to promote HIV-1 infection may be offset by the antiviral actions of a variety of ISGs.

Interestingly, previous studies demonstrated that the effect of IFN-α is different during the acute and chronic phases of infection in SIV-infected macaques [72] and HIV-1-infected humanized mouse models [73,74]. During the acute phase of infection, IFN-α suppresses viral replication by inducing antiviral ISG expression [73,74]. On the other hand, the IFN-α responses during the chronic phase of infection potentially exacerbate disease progression [72,74]. These findings suggest that the IFN-α effect and the IFN-α-associated viral phenotypes are different depending on the phases of infection *in vivo*. Here we demonstrated that cell-to-cell infection of TF virus is resistant to IFN-α-mediated antiviral effect. A major difference between the TF and CC viruses appears to be the ability to replicate in the shaking culture, which only allows cell-free infection, without IFN-α, while CC virus depends on the enhancement of cell-free infection by IFN-α. Based on our findings and previous insights, we can consider the following scenario (**Fig 8B**): since cell-to-cell infection (*R*_*cc*_) dominantly contributes to HIV-1 infection compared to cell-free infection (*R*_*cf*_), our findings suggest that the resistance of TF viruses against the IFN-α-mediated antiviral effect [5,6,8] is due to the resistance to inhibition of cell-to-cell infection by IFN-α. In fact, cell-to-cell HIV-1 infection is a notable mode of infection during the acute phase of infection *in vivo* for multiple reasons. First, in humanized mouse models, cell-to-cell HIV-1 infection has been visualized in the draining lymph nodes using two-photon microscopy [75,76] and the gut-associated lymphoid tissues using the electron tomography [77]. Second, cell-to-cell infection in the local tissues such as exposed mucosa (e.g., genital tracts) is crucial for viral spread [78,79]. Collectively, cell-to-cell HIV-1 infection appears to be a crucial step during the acute phase of infection *in vivo*, and it strongly suggests that rendering resistance to IFN-α-mediated blockage of cell-to-cell infection is important for successful viral spread. Importantly, IFITMs impair cell-to-cell HIV-1 infection [69] and TF viruses are resistant to the antiviral effect mediated by IFITMs [34]. These findings suggest that the resistance to IFN-α-mediated inhibition of cell-to-cell infection is an important phenotype for TF viruses to establish infection in individuals during acute HIV-1 infection (**Fig 8B, top**).

In contrast to the resistance of TF virus to the IFN-α-mediated inhibition of cell-to-cell infection, IFN-α conferred a promotive effect on the cell-free infection of CC virus. We also showed that an ISG, LY6E, has the ability to promote cell-free infection. During the chronic phase of infection, it might be beneficial for HIV-1 to accept the IFN-α-mediated promotion of cell-free infection rather than to tolerate the IFN-α-mediated inhibition of cell-to-cell infection. In lymphoid tissues, CD4^+^ T cells and viral particles are locally packed at a high density [80]. Therefore, the parameter *V/C* in the lymphoid tissues in individuals exhibiting high viremia should be relatively high compared to those in blood and *in vitro* cell culture conditions. In fact, viral particles are detected in the lymphoid tissues of HIV-1-infected individuals or SIV-infected macaques [80–83] and *LY6E* expression is up-regulated in the CD4^+^ T cells of chronically infected individuals [52–55]. Therefore, CC viruses may have been selected to be able to utilize the ISGs, including LY6E, that potently promote cell-free infection (**Fig 8B, bottom**). Furthermore, neutralizing antibodies (Nabs) emerge in individuals during chronic infection, and both cell-to-cell and cell-free infections can be impaired by Nabs [84]. More importantly, there is a trade-off for HIV-1 to be resistant against the two antiviral effects, IFITMs and Nabs [34]. Although TF viruses are resistant to IFITMs, HIV-1 loses the resistant ability to IFITMs in order to escape from the Nab-mediated antiviral effect [34]. Therefore, the conflict on the resistance to either IFITMs or Nabs (i.e., the gain of the resistance to Nab-mediated antiviral effect leads to the loss of the resistance to the IFITM-mediated antiviral effect) may be a driving force to switch IFN-α-associated viral phenotype. Altogether, our findings suggest that, through the course of infection in individuals, the IFN-α-associated HIV-1 phenotype shifts from the tolerance to IFN-α-mediated inhibition of cell-to-cell infection (the acute phase of infection by TF viruses) to the susceptibility to IFN-α-mediated promotion of cell-free infection (the chronic phase of infection by CC viruses) (**Fig 8B**). A limitation of our study is that we used only a pair of TF and CC viruses (CH077). To fully elucidate the differences of the virological properties of TF and CC viruses and their interplay with IFN-α, further studies using a variety of TF/CC pairs will be needed. Nevertheless, through the experimental-mathematical approach used here we provide evidence suggesting that the difference in the phenotype of TF and CC viruses with respect to their IFN sensitivity can explain their strategies to adapt to the infected hosts.

## Methods

### Cell culture and HIV-1 infection

Three human CD4^+^ T cell lines, Jurkat [85], Jurkat-CCR5 [86] and SupT11-CCR5 [87–89] cell lines, were cultured with RPMI 1640 (Sigma) containing 2% fetal calf serum (FCS) and antibiotics (penicillin and streptomycin) as previously described [19]. The growth speed of the cultured cells with 2% FCS was ~30% of that with 10% FCS. Note that the SupT11 cell line is a permissive clone derived by limiting dilution from SupT1 [89] and its sensitivity to IFN-α is comparable to parental SupT1 cells (personal communication). Primary human CD4^+^ T cells were prepared as previously described [73,90]. Briefly, human peripheral mononuclear cells (PBMCs) were isolated from human peripheral blood using Ficoll-Paque (Pharmacia) and human CD4^+^ T cells were isolated human CD4^+^ T cell isolation kit (Miltenyi) according to the manufacturers’ protocol. These cells were activated with anti-CD3/anti-CD28 dynabeads (Thermo Fisher Scientific) and maintained in RPMI1640 (Sigma) containing 10% FCS and antibiotics (penicillin and streptomycin) with human interleukin-2 (100 U/ml).

The virus solution was prepared as previously described [19,91–93]. Briefly, 30 μg of HIV-1 plasmids, strains NL4-3 (GenBank accession no. M19921.2), CH077_CC [94] and CH077_TF (GenBank accession no. JN944941.1 [95]) (kindly provided by Daniel Sauter and Frank Kirchhoff) were transfected into 293T cells by the calcium-phosphate method. At 48 h post-transfection, the culture supernatant was harvested, centrifuged, and then filtered through a 0.45-μm-pore-size filter to produce virus solution. The infectivity of virus solution was titrated as previously described [19]. Briefly, the virus solution obtained was serially diluted and then inoculated onto phytohemagglutinin-stimulated human peripheral blood mononuclear cells in a 96-well plate in triplicate. At 14 days postinfection, the endpoint was determined by using an HIV-1 p24 antigen enzyme-linked immunosorbent assay (ELISA) kit (ZetptoMetrix) according to the manufacture’s procedure, and virus infectivity was calculated as the 50% tissue culture infectious doses (TCID_50_) according to the Reed-Muench method.

HIV-1 infection was performed as previously described [19]. Briefly, 3 × 10^5^ of Jurkat cells or Jurkat-CCR5 were infected with HIV-1 (multiplicity of infection [MOI] 0.1 for NL4-3 and 0.05 for CH077_CC and CH077_TF) at 37°C for 2 hr. The infected cells were washed three times with the culture medium, and then suspended with 3 ml of culture medium and seeded into a 25-cm^2^ flask (Nunc). For the culture with IFN-α treatment, the cells were pre-treated with IFN-α (100 U/ml, Sigma. Cat# SRP4596-100UG) at 24 h before infection, and were maintained in the presence of IFN-α (100 U/ml) throughout the experiments. For the static infection, the infected cell culture was kept in a 37°C/5% CO_2_ incubator as usual. For the shaking infection, the infected cell culture was handled as previously described [19,21]. Briefly, the cell culture was kept on a Petit rocker Model-2230 (a rocking shaker; Wakenyaku) placed in 37°C/5% CO_2_ incubator, and was gently shaken at 40 movements per min. The amount of virus particles in the culture supernatant and the number of infected cells were measured at the indicated timepoints as follows: a portion (300 μl) of the infected cell culture was harvested, and the amount of released virions in the culture supernatant was quantified by using an HIV-1 p24 antigen ELISA kit (ZetptoMetrix) according to the manufacture’s procedure. The cell number was counted by using a Scepter handled automated cell counter (Millipore) according to the manufacture’s protocol. The percentage of infected cells was measured by flow cytometry. The detection threshold of each value are the followings: cell number (cell counting), 3,000 cells/ml; % p24-positive cells (flow cytometry), 0.3%; and p24 antigen in culture supernatant (p24 antigen ELISA), 80 pg/ml. The remaining cell culture was centrifuged and then resuspended with 3 ml of fresh culture medium with or without IFN-α.

### Quantification of Jurkat cell growth

To quantify the growth kinetics of Jurkat cells, we used the following mathematical model:

$$\frac{dT(t)}{dt}=gT(t)[1-\frac{T(t)}{T_{max}}].$$

Here the variable *T*(*t*) is the number of Jurkat cells at time *t* and the parameters *g* and *T*_*max*_ are the maximum growth rate of the cells and the carrying capacity of the cell culture flask, respectively. Nonlinear least-squares regression (*constRoptim* package of *R*) was performed to fit the above equation to the time-course numbers of Jurkat cells for each experimental condition (i.e. static and shaking culture without and with IFN-α). Note that *T*_*max*_ is assumed to be common among experimental conditions. The fitted parameter values are listed in **S2 Table** and the model behavior using these best-fit parameter estimates is presented together with the data in **S2 Fig**.

### Flow cytometry

Flow cytometry was performed with a FACSCalibur (BD Biosciences) as previously described [19,91–93]. To detect viral p24 antigen, a FITC-labeled anti-HIV-1 p24 monoclonal antibody (KC57; Beckman Coulter) was used. To analyze the expression levels of CD4 and CCR5 on Jurkat-CCR5 cells and its derivatives (see below), a FITC-labeled anti-CD4 monoclonal antibody (RPA-T4; Biolegend) and a PE-labeled anti-CCR5 monoclonal antibody (2D7; BD Biosciences) were used. The obtained data were analyzed with CellQuest software (BD Biosciences).

### Data fitting and parameter estimation

To assess the variability of kinetic parameters and model prediction, we perform Bayesian estimation for the whole dataset using MCMC sampling as previously described [19]. In the Bayesian inference, it is assumed that measurement error obeys normal distribution with mean zero and unknown variance (i.e., error variance), and the error variance follows the Gamma distribution as its prior distribution. Posterior predictive parameter distributions were estimated as outcome of MCMC computation. We also simultaneously fit Model 0 (or 1) with *ω*>0 and *ω* = 0 to the concentration of p24-negative and p24-positive target cells and the amount of p24 viral protein in the static and shaking cell cultures without and with IFN-α, respectively. Using the last 100,000 among 150,000 accepted MCMC parameter estimates from the time-course experimental datasets, we calculated the basic reproduction numbers. Note that the basic reproduction numbers in Model 0 derived in our previous study [19] are corresponding to those in Model 1 because the linearized equations of Models 0 and 1 are the same.

### Calculation of *V/C* value

It is estimated that an HIV-1 viral particle contains approximately 5,000 Gag molecules [96,97]. This indicate that a viral particle contains approximately 5,000 p24 molecules. The molecular weight of p24 is 23.81 kDa, thereby the weight of p24 per virion was calculated as follows: 2.381×10^4^ (g/mol) ×5.0×10^5^ (molecules)/6.022×10^33^ (molecules/mol) = 1.98×10^−16^ (g). By using this value, the number of viral particles was calculated from the amount of p24. We defined *V/C* as the parameter for the density of cells and viruses. *V/C* is calculated as the ratio of the number of viral particles to the number of cells in a culture divide by the culture volume as illustrated in **Fig 3**.

### Single round infection assay

Jurkat-CCR5, SupT11-CCR5 cells and primary human CD4^+^ T cells (3.0 × 10^5^ cells) were seeded with or without IFN-α (100 U/ml) and NL4-3 (containing 818 ng p24, which provides ~10% of p24-positive cells at 48 h postinfection by flow cytometry) was inoculated into the culture medium whose final volumes were 500 μl (in 24-well plate), 1 ml (in 24-well plate), 2 ml (in 6-well plate), 3 ml (in 25-cm^2^ flask), and 6 ml (in 25-cm^2^ flask), respectively. For the culture with IFN-α treatment, the cells were pre-treated with IFN-α (100 U/ml) at 24 h before infection. Without washing viruses out, the infected cell cultures were kept incubated in 37°C/5% CO_2_ incubator. After 48 h postinfection, the cells were collected and the percentage of infected cells was measured as described above.

### Preparation of LY6E-HA-expressing cells

The open reading frame (ORF) of LY6E-HA was obtained as previously described [41]. Briefly, to add the HA tag in LY6E ORF, overlap extension PCR was performed by using a LY6E expression vector pSCRPSY-LY6E (a kind gift from Sam Wilson) and the following primers: for 1st PCR, first half, 5’-ccc cgg ggg aat tcA TGA AGA TCT TCT TGC CAG TGC TGC TGG-3’; and 5’-ACT AGC GTA ATC TGG AAC ATC GTA TGG GTA GAA ATT GCA CAG AAA GCT CTG GCA GCA-3’. For 1st PCR second half, 5’-TTC TAC CCA TAC GAT GTT CCA GAT TAC GCT AGT GCG GCC GAT GGC GGG CT-3’; and, 5’-aat taa ttg cgg ccg cTC AGG GGC CAA ACC GCA GCA-3’. For 2nd PCR, 5’-ccc cgg ggg aat tcA TGA AGA TCT TCT TGC CAG TGC TGC TGG-3’; and 5’-aat taa ttg cgg ccg cTC AGG GGC CAA ACC GCA GCA-3’. Then, the LY6E-HA ORF was inserted into pCSII-CMV-MCS-IRESII-Blastcidin as previously described [73]. Briefly, the LY6E-HA ORF and pCSII-CMV-MCS-IRESII-Blastcidin were digested with EcoRI and NotI and the digested fragments were ligased by using T4 Ligase (New England Biolabs). The product sequence was confirmed by Sanger sequence (Fasmac).

The lentiviral vector expressing LY6E-HA was prepared as previously described [73]. Briefly, the LY6E-HA-expressing plasmid or empty vector (pCSII-CMV-MCS-IRESII-Blastcidin, as a negative control) was co-transfected with pCAG-HIVgp and pCMV-VSV-G-RSV-Rev [98] into HEK293T cells by the calcium-phosphate method. At 48 h post-transfection, the culture supernatant was harvested, centrifuged, and filtered through a 0.45-μm-pore-size filter. The collected lentiviral vectors were transduced into Jurkat-CCR5 and SupT11-CCR5 cells, and the transduced cells were selected by 10 μg/ml blastcidin (Sigma).

### Preparation of *LY6E* KO cells

*LY6E* KO and control Jurkat-CCR5 cells were generated by transduction with blasticidin-selectable lentiCRISPRv2 expressing non-targeting sgRNA (sgNT) or *LY6E*-targeting sgRNA. Guide RNA sequences were cloned into the lentiCRISPRv2 blast plasmid (a gift from Mohan Babu [Addgene plasmid #83480; http://n2t.net/addgene:83480; RRID: Addgene_83480]) as previously described 25075903. The following guide sequences were used to target *LY6E*: 5’-cac cgA TTG CTC TTC TGG TTC AAG C-3’ and 5’-aaa cGC TTG AAC CAG AAG AGC AAT c-3’. The following non-targeting guide sequences were used: 5’-cac cgT GTT CTA CTT TCG AAG TTA A-3’ and 5’-aaa cTT AAC TTC GAA AGT AGA ACA c-3’ [99]. HEK293T were transfected with plasmids expressing 1) plentiCRISPRv2-blast-sgLY6E or plentiCRISPRv2-blast-sgNT, 2) HIV gag-pol, and 3) VSV-G in a ratio of 1:0.7:0.5 with X-tremeGENE 9 (Sigma) in DMEM with 10% FCS and antibiotics (penicillin and streptomycin). Transfection media was aspirated at 6 hours post-transfection and replaced with fresh media. Lentivirus-containing supernatant was collected at 48 and 72 h, cleared by centrifugation, supplemented with 20 μg/mL polybrene, and used immediately for transduction. Transduced cells were recounted 48 h post-transduction and selected in RPMI with 10% FCS, antibiotics (penicillin and streptomycin), puromycin (0.5 μg/ml), and blasticidin (10 μg/ml). Cells were passaged for 29 days in selection media.

### Western blotting

To verify LY6E-HA expression in the transduced cells, Western blotting was performed as described previously [73]. Briefly, 1.0 × 10^6^ cells were lysed with RIPA buffer (50 mM Tris-HCl buffer [pH 7.6], 150 mM NaCl, 1% Nonidet P-40, 0.5% sodium deoxycholate, 0.1% SDS) with protease inhibitor cocktail (Roche). Proteins in lysate were separated by SDS-PAGE with 14% acrylamide gel and then transferred to PVDF membrane. The following antibodies were used for protein detection: anti-HA antibody (3F10; Roche), anti-alpha-tubulin (TUBA) antibody (DM1A; Sigma). To verify the expression of endogenous LY6E in LY6E KO Jurkat-CCR5 cells, *LY6E* KO and control cells were treated with IFN-α (100 U/ml) for 24 h and then lysed in RIPA buffer. Lysates were processed and Western blotting for endogenous LY6E was performed as previously described with modifications as described below [41]. Briefly, proteins were separated on a low molecular weight tricine gel and transferred to PVDF membrane. LY6E was detected using a mouse anti-LY6E monoclonal antibody (4D8.6.7, Genentech), and beta-actin (ACTB) was detected using peroxidase-conjugated anti-ACTB monoclonal antibody (AC-15, Sigma).

### RNA-Seq and transcriptome analysis

Cellular RNA was extracted from Jurkat cells with or without IFN-α treatment for 24 h (100 U/ml) by using RNeasy Mini kit (Qiagen) according to the manufacture’s protocol. RNA-Seq was conducted in Medical and Biological Laboratories, Co. (Nagoya, Japan) as previously described [73,90]. Raw sequence reads data were mapped to the human reference genome (hg38) by using Tophat2 v2.1.1 [100] in the default setting. The raw read count matrix was obtained by using featureCounts v1.5.3 with the annotation. The raw read count was normalized by DESeq2 v1.18 [101] and the data were classified into the four conditions: static culture without IFN-α, static culture with IFN-α, shaking culture without IFN-α, and shaking culture with IFN-α. For comparison of gene expression levels among samples, reads per kilobase of exon per million mapped reads (RPKM) of each gene was calculated as follows: the number of reads mapped to exons of each gene was normalized by dividing the sum of read counts among all genes in the sample in millions to calculate RPM (reads per million mapped reads). Then RPM value was normalized by dividing the sum of exon length in kilobases. Then, the data were analyzed to detect the DEGs either by IFN-α treatment or shaking culture condition by using DESeq2 v1.18 [101]. The DEGs were selected by the following criteria: (1) the false discovery rate (FDR) calculated by the Benjamin-Hochberg method is less than 0.01; and (2) the absolute value of median of log2 fold change is more than 0.5. The clustering analysis was performed by using all expressed genes. Functional annotation for the DEGs was carried out by using clusterProfiler package v3.6 [102], and the DEGs were annotated to Gene Ontology Biological Process (GOBP) gene set. The *p* value adjustments for multiple comparisons were performed by the Benjamin-Hochberg method.

### Statistical analysis

For the data shown in **Figs 4, 6B–6E, 7B–7E** and **S3B–S3E**, we conducted bootstrap Brunner Munzel test to assess if the two probability distributions have significant difference [103]. We applied the test for the distribution of *R*_0_, *R*_*cf*_, *R*_*cc*_ without and with IFN-α. In total 200,000 parameter sets were sampled with replacement from the posterior predictive distribution to calculate summary statistics of Brunner Munzel for *R*_0_, *R*_*cf*_, *R*_*cc*_. We repeated this 100 times repeatedly to avoid potential bias due to sampling. The average *p* values were used as an indicator of the difference in distributions without and with IFN-α.

Unless otherwise stated, data analyses were performed using GraphPad Prism software. The experimental data (**Figs 3C, 7C** and **S4**) are presented as averages ± SEM, and the statistically significant differences were determined by Student’s *t* test.

## Supporting information

S1 Fig No effect of the shaking procedure on cell-free infection. Jurkat cells were infected with HIV-1 (at multiplicity of infection 1) as described in **Methods**, and the infected cells were cultured in the static and the shaking condition. By harvesting the cells at 24 and 48 hours postinfection, the cells were analyzed by flow cytometry as described in **Methods**. The percentage of the average of p24-positive cells are shown with SD. The assay was performed in triplicate, and the representative result is shown. This data is the same as supplement figure 1 in our previous study [19].
(JPG)

S2 Fig Dynamics of Jurkat cell growth in the static and shaking culture with or without IFN-α treatment. By harvesting the cells in the static culture without IFN-α (**a**), in the shaking culture without IFN-α and (**b**), in the static culture with IFN-α (**c**), and in the shaking culture with IFN-α (**d**), the growth kinetics in three independent experimental replicates for each condition was estimated as described in Methods.
(TIF)

S3 Fig Dynamics of HIV-1 NL4-3 infection through cell-to-cell and cell-free infection without or with IFN-α (Model 0). Jurkat cells were infected with HIV-1 strain NL4-3 at MOI 0.1 in the static and shaking culture under treated with IFN-α or untreated. **a** Time-course experimental data and the fitting of Model 0 (see Methods). The time-course of experimental data for the numbers of uninfected cells (top) and infected cells (middle), and the amount of viral p24 antigen in the culture supernatant (bottom) in the static culture (left) and shaking culture (right) are shown. Gray and orange curves respectively indicate the results in the absence or presence of IFN-α treatment. The dots with error bars are the averages and SEMs of three independent experiments. The shadow regions correspond to 95% posterior predictive intervals, and the curves give the best-fit solution Model 0 to the experimental dataset. **b-e** The comparison of basic reproduction numbers in the absence or presence of IFN-α. (Left) The distributions of *R*_0_ (**b**), *R*_*cf*_ (**c**), and *R*_*cc*_ (**d**), computed from the all accepted parameters by MCMC methods, are shown. The contribution of the cell-to-cell infection (i.e. *R*_*cc*_/(*R*_*cc*_+*R*_*cf*_)) is shown in panel **e**. In these analyses, we sampled the 20,000 parameter sets from MCMC computation among 150,000 samples. For detail, please see Methods. (Right) The bars indicate the mean values computed by MCMC methods. Orange and gray respectively indicate the data with and without IFN-α treatment. The p values are calculated by Brunner Munzel test.
(TIF)

S4 Fig Promotion of cell-free HIV-1 infection by IFN-α under high cell-virus density in SupT11 cells. Single-round infection assay under different density conditions. The *V/C* at different five culture conditions (0.5, 1, 2, 3, and 6 ml) corresponds to that shown in **Fig 3A**. Single round infection assay were performed at five different cell-virus densities with or without IFN-α. Each dot indicate the result from one culture, and three independent experiments was performed. The bars with error bars are the averages and SEMs of three independent experiments. Asterisks indicate statistically significant differences were determined by Student’s *t* test (*p*<0.05).
(TIF)

S5 Fig Differential gene expression profile of Jurkat cell by IFN-α. **a** tSNE clustering for the gene expression profile of Jurkat cells in static (circle) or shaking (diamond) cultures and with or without IFN-α. Orange and gray respectively indicate the data with and without IFN-α treatment. Each dot represents one RNA-seq dataset. Three experimental replicates per each condition were prepared. **b** The expression levels of seven anti-HIV-1 ISGs. Each symbol indicate the result from one replicate, and the bars with error bars indicate the averages with SEMs of three independent replicates. RPKM, reads per kilobase of exon per million mapped reads. The lists of all DEGs are shown in **S7** and **S8 Tables**.
(TIF)

S6 Fig Surface expression of CD4 and CCR5 on Jurkat-CCR5 cells expressing LY6E-HA. Surface expression levels of CD4 (x-axis) and CCR5 (y-axis) on parental Jurkat-CCR5 cells (left), empty vector-transduced cells (middle), and LY6E-HA-transduced cells (right) were analyzed by flow cytometry. Representative results are shown.
(TIF)

S7 Fig Surface expression of CD4 and CCR5 on *LY6E* KO Jurkat-CCR5 cells. Surface expression levels of CD4 (x-axis) and CCR5 (y-axis) on non-target (NT) gRNA-transduced Jurkat-CCR5 cells (left) and *LY6E* KO cells (right) were analyzed by flow cytometry. Representative results are shown.
(TIF)

S1 Table Estimated parameters fitting the experimental data of HIV-1 strain NL4-3 by Model 0. (DOCX)

S2 Table Estimated parameters and the initial values for Jurkat cell growth. (DOCX)

S3 Table The estimated initial values for HIV-1 strain NL4-3 by Model 0. (DOCX)

S4 Table The estimated initial values for HIV-1 strain NL4-3 by Model 1. (DOCX)

S5 Table List of DEGs by IFN-α treatment. (XLSX)

S6 Table List of DEGs under shaking culture condition. (XLSX)

S7 Table List of gene ontology of the DEGs by IFN-α treatment. (XLSX)

S8 Table The estimated initial values for HIV-1 strain CH077_CC by Model 1. (DOCX)

S9 Table The estimated initial values for HIV-1 strain CH077_TF by Model 1. (DOCX)
