# Supplementary figures and images for: Antithetic effect of interferon-α on cell-free and cell-to-cell HIV-1 infection
Source: PLoS Comput Biol. 2022 Apr 25;18(4):e1010053. doi: 10.1371/journal.pcbi.1010053 (PMC9037950; doi:10.1371/journal.pcbi.1010053)

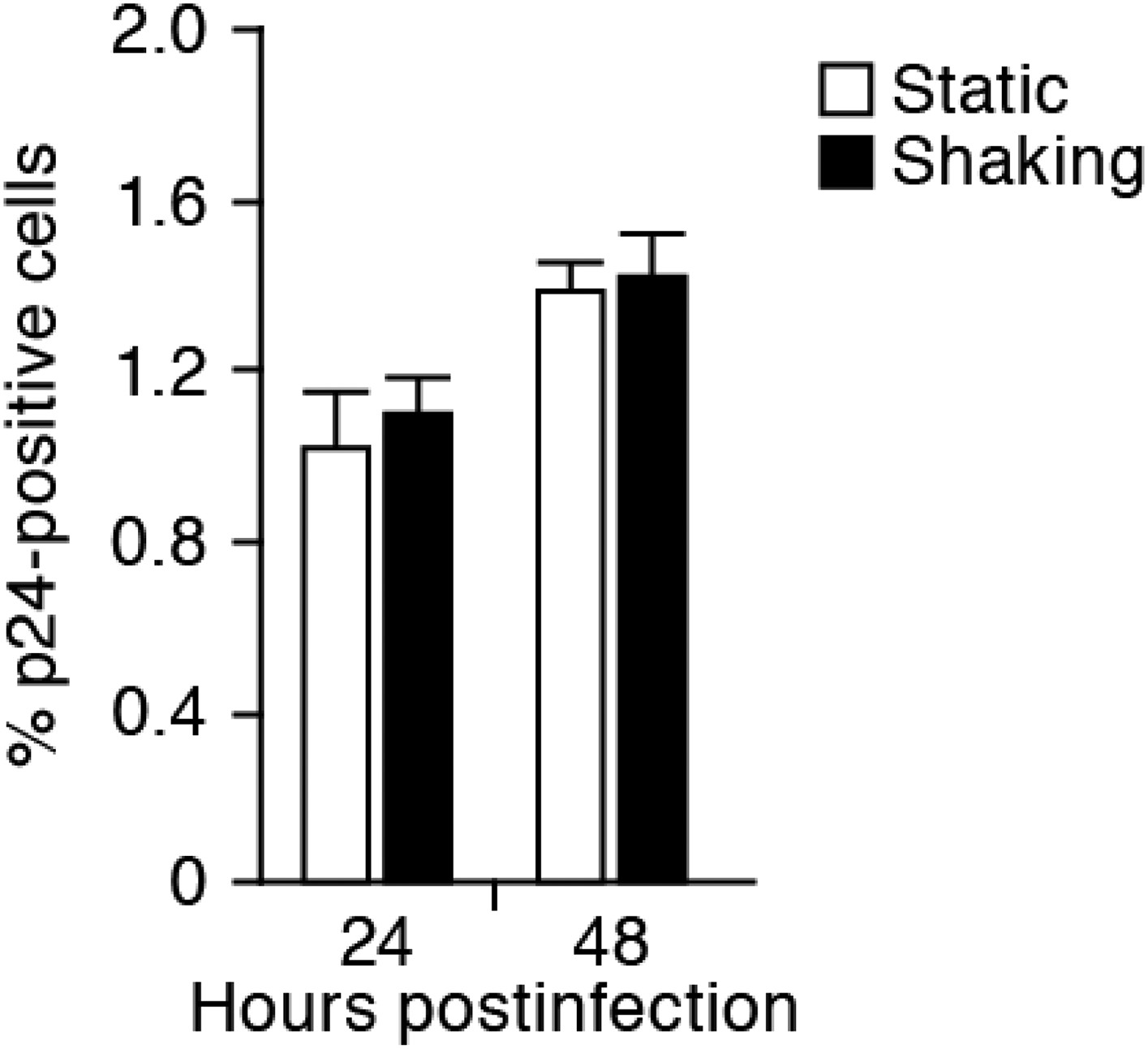

Supplement: S1 Fig — Jurkat cells were infected with HIV-1 (at multiplicity of infection 1) as described in Methods, and the infected cells were cultured in the static and the shaking condition. By harvesting the cells at 24 and 48 hours postinfection, the cells were analyzed by flow cytometry as described in Methods. The percentage of the average of p24-positive cells are shown with SD. The assay was performed in triplicate, and the representative result is shown. This data is the same as supplement figure 1 in our previous study [19]. (JPG) [file pcbi.1010053.s001.jpg]

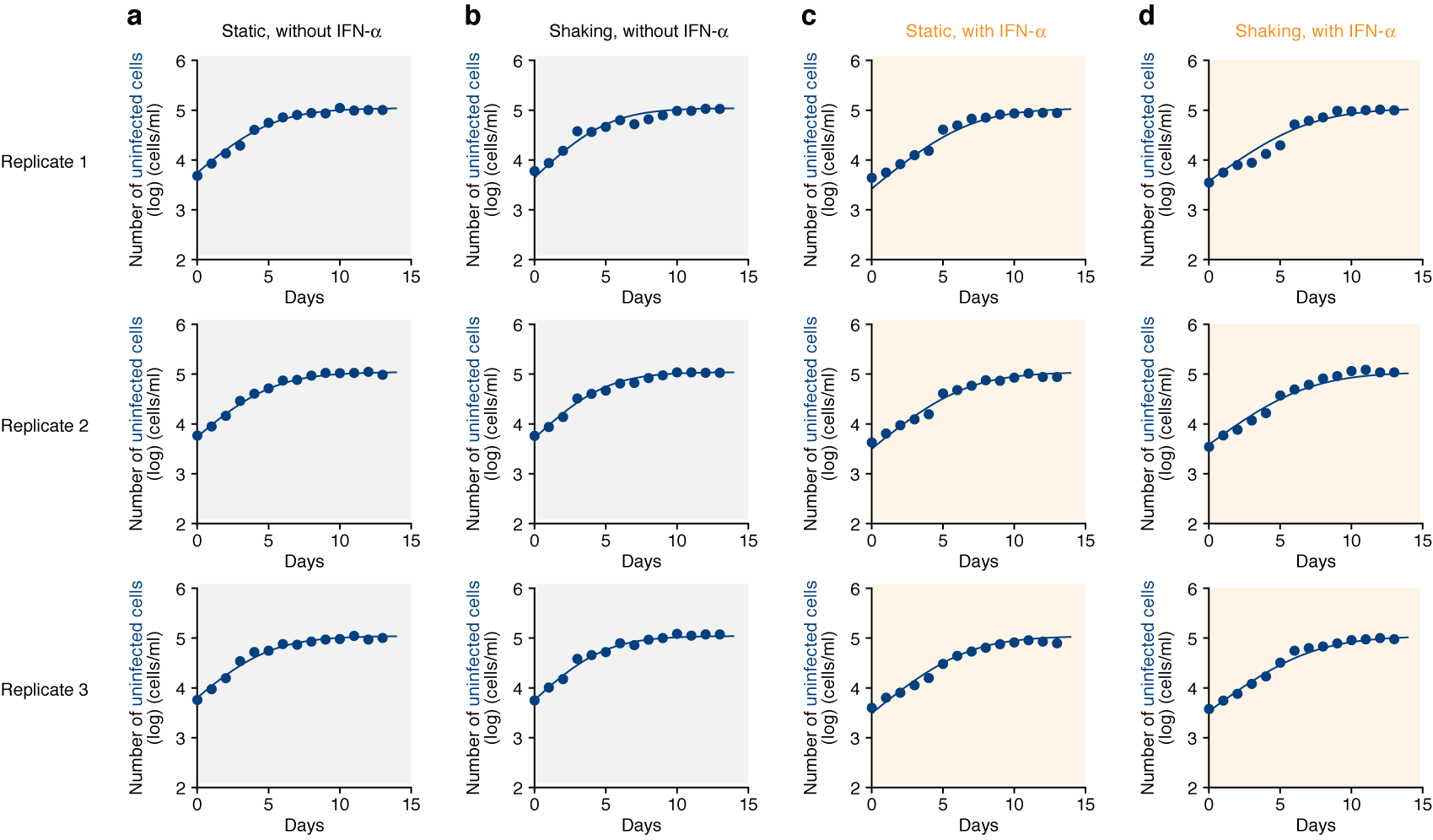

Supplement: S2 Fig — By harvesting the cells in the static culture without IFN-α (a), in the shaking culture without IFN-α and (b), in the static culture with IFN-α (c), and in the shaking culture with IFN-α (d), the growth kinetics in three independent experimental replicates for each condition was estimated as described in Methods. (TIF) [file pcbi.1010053.s002.tif]

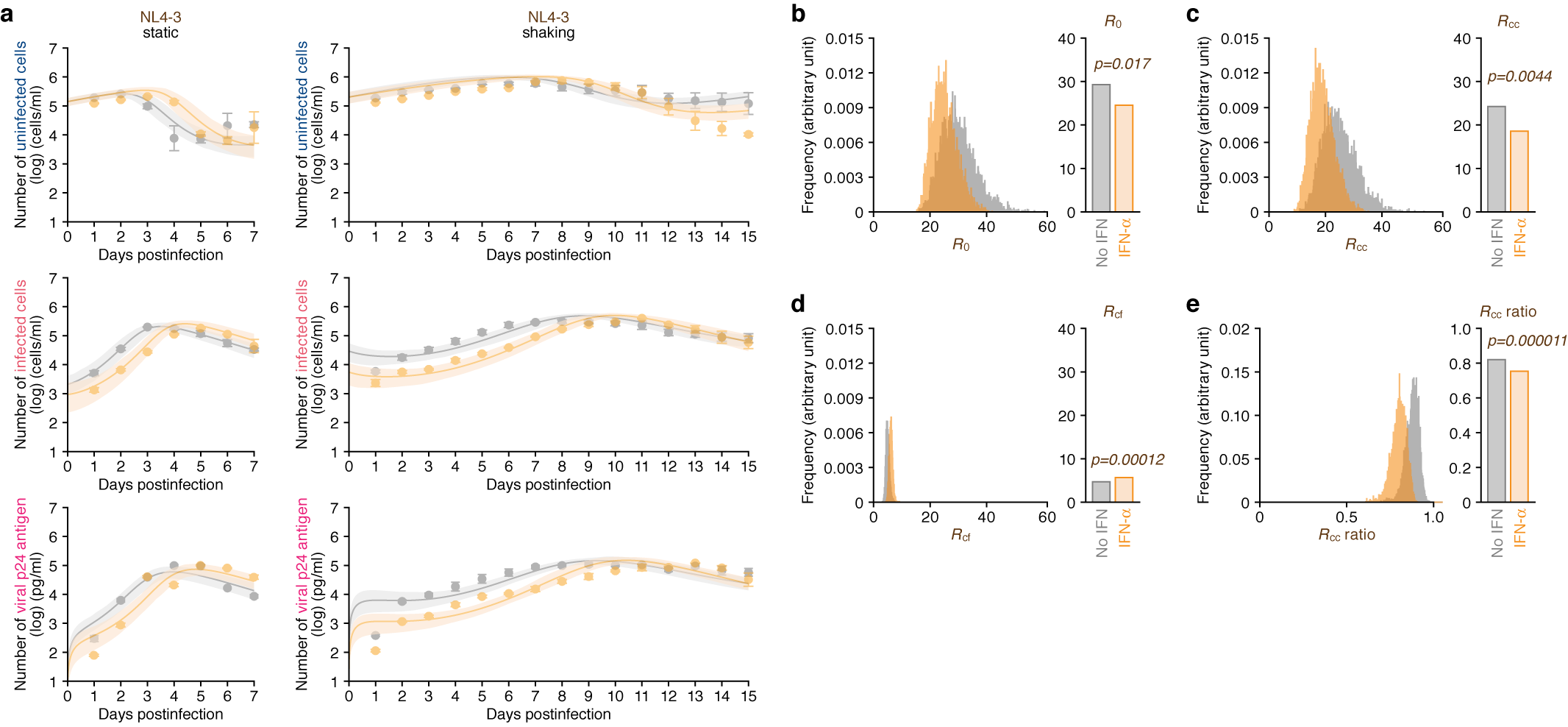

Supplement: S3 Fig — Jurkat cells were infected with HIV-1 strain NL4-3 at MOI 0.1 in the static and shaking culture under treated with IFN-α or untreated. a Time-course experimental data and the fitting of Model 0 (see Methods). The time-course of experimental data for the numbers of uninfected cells (top) and infected cells (middle), and the amount of viral p24 antigen in the culture supernatant (bottom) in the static culture (left) and shaking culture (right) are shown. Gray and orange curves respectively indicate the results in the absence or presence of IFN-α treatment. The dots with error bars are the averages and SEMs of three independent experiments. The shadow regions correspond to 95% posterior predictive intervals, and the curves give the best-fit solution Model 0 to the experimental dataset. b-e The comparison of basic reproduction numbers in the absence or presence of IFN-α. (Left) The distributions of R0 (b), Rcf (c), and Rcc (d), computed from the all accepted parameters by MCMC methods, are shown. The contribution of the cell-to-cell infection (i.e. Rcc/(Rcc+Rcf)) is shown in panel e. In these analyses, we sampled the 20,000 parameter sets from MCMC computation among 150,000 samples. For detail, please see Methods. (Right) The bars indicate the mean values computed by MCMC methods. Orange and gray respectively indicate the data with and without IFN-α treatment. The p values are calculated by Brunner Munzel test. (TIF) [file pcbi.1010053.s003.tif]

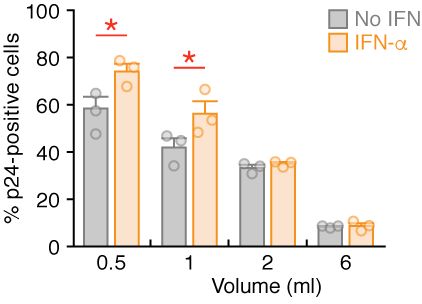

Supplement: S4 Fig — Single-round infection assay under different density conditions. The V/C at different five culture conditions (0.5, 1, 2, 3, and 6 ml) corresponds to that shown in Fig 3A. Single round infection assay were performed at five different cell-virus densities with or without IFN-α. Each dot indicate the result from one culture, and three independent experiments was performed. The bars with error bars are the averages and SEMs of three independent experiments. Asterisks indicate statistically significant differences were determined by Student’s t test (p<0.05). (TIF) [file pcbi.1010053.s004.tif]

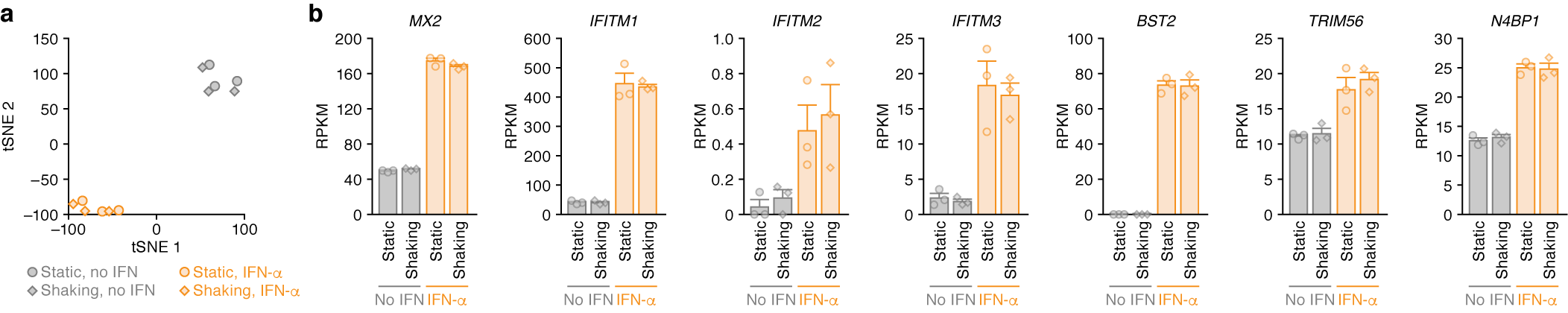

Supplement: S5 Fig — a tSNE clustering for the gene expression profile of Jurkat cells in static (circle) or shaking (diamond) cultures and with or without IFN-α. Orange and gray respectively indicate the data with and without IFN-α treatment. Each dot represents one RNA-seq dataset. Three experimental replicates per each condition were prepared. b The expression levels of seven anti-HIV-1 ISGs. Each symbol indicate the result from one replicate, and the bars with error bars indicate the averages with SEMs of three independent replicates. RPKM, reads per kilobase of exon per million mapped reads. The lists of all DEGs are shown in S7 and S8 Tables. (TIF) [file pcbi.1010053.s005.tif]

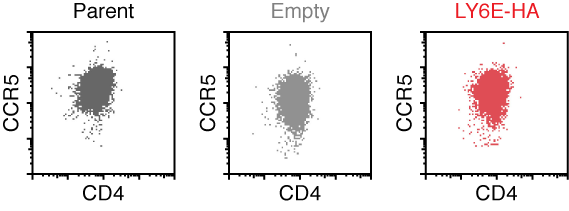

Supplement: S6 Fig — Surface expression levels of CD4 (x-axis) and CCR5 (y-axis) on parental Jurkat-CCR5 cells (left), empty vector-transduced cells (middle), and LY6E-HA-transduced cells (right) were analyzed by flow cytometry. Representative results are shown. (TIF) [file pcbi.1010053.s006.tif]

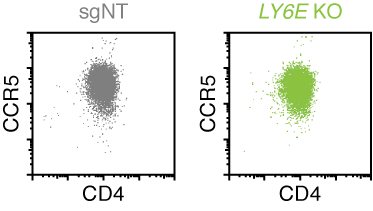

Supplement: S7 Fig — Surface expression levels of CD4 (x-axis) and CCR5 (y-axis) on non-target (NT) gRNA-transduced Jurkat-CCR5 cells (left) and LY6E KO cells (right) were analyzed by flow cytometry. Representative results are shown. (TIF) [file pcbi.1010053.s007.tif]
