# Supplementary material for: Antithetic effect of interferon-α on cell-free and cell-to-cell HIV-1 infection
Source: PLoS Comput Biol. 2022 Apr 25;18(4):e1010053. doi: 10.1371/journal.pcbi.1010053 (PMC9037950; doi:10.1371/journal.pcbi.1010053)
Supplement: S1 Table — (DOCX) [file pcbi.1010053.s008.docx]

**S1 Table. Estimated parameters fitting the experimental data of HIV-1 strain NL4-3 by Model 0.**

| Parameter name | Symbol | Unit | Without IFN-α | | With IFN-α | |
| --- | --- | --- | --- | --- | --- | --- |
|  |  |  | Mean | 95% CI* | Mean | 95% CI* |
| Rate constant for cell-free infection | $\beta$ | ${10}^{-6}\times$(p24 day)^-1^ | 6.276 | 4.480 – 8.285 | 7.393 | 6.156 – 8.726 |
| Rate constant for cell-to-cell infection | $\omega$ | ${10}^{-6}\times$(cell day)^-1^ | 9.495 | 6.158 – 13.86 | 7.346 | 4.749 – 10.76 |
| Death rate of infected cells | $\delta$ | day^-1^ | 0.6977 | 0.5728 – 0.8364 | 0.6977 | 0.5728 – 0.8364 |
| Production rate of total viral protein | $p$ | day^-1^ | 0.7146 | 0.5196 – 0.9602 | 0.7146 | 0.5196 – 0.9602 |
| Basic reproduction number through cell-to-cell infection | $R_{cc}$ ($=\frac{\omega K}{\delta}$) | --- | 24.50 | 15.16 – 37.23 | 18.91 | 11.91 – 28.42 |
| Basic reproduction number through cell-free infection | $R_{cf}$ ($=\frac{p\beta K}{c\delta}$) | --- | 4.905 | 3.864 – 6.134 | 5.823 | 4.706 – 7.111 |
| Basic reproduction number | $R_{0}$ ($=R_{cc}+R_{cf}$) | --- | 29.41 | 19.84 – 42.32 | 24.74 | 17.58 – 34.41 |
| Contribution of cell-to-cell infection | $\frac{R_{cc}}{R_{cc}+R_{cf}}$ | --- | 0.8279 | 0.7526 – 0.8868 | 0.7586 | 0.6650 – 0.8344 |

*CI: credible interval.
