# Supplementary material for: Antithetic effect of interferon-α on cell-free and cell-to-cell HIV-1 infection
Source: PLoS Comput Biol. 2022 Apr 25;18(4):e1010053. doi: 10.1371/journal.pcbi.1010053 (PMC9037950; doi:10.1371/journal.pcbi.1010053)
Supplement: S2 Table — (DOCX) [file pcbi.1010053.s009.docx]

**S2 Table. Estimated parameters and the initial values for Jurkat cell growth.**

| Parameter Name | Symbol | Unit | Experiment 1 | Experiment 2 | Experiment 3 |
| --- | --- | --- | --- | --- | --- |
| NL4-3 (model 0) | | | | | |
| Growth rate of Jurkat cells in shaking cell culture without IFN-α | $g$ | day^-1^ | 0.5174 | | |
| Growth rate of Jurkat cells in static cell culture for without IFN-α |  |  | 0.4754 | | |
| Growth rate of Jurkat cells in shaking cell culture with IFN-α |  |  | 0.3978 | | |
| Growth rate of Jurkat cells in static cell culture with IFN-α |  |  | 0.4351 | | |
| Initial number of Jurkat cells in shaking cell culture for without IFN-α | $T(0)$ | ${10}^{4}\times$cells/ml | 16.08 | 18.03 | 19.34 |
| Initial number of Jurkat cells in static cell culture for without IFN-α |  |  | 18.94 | 18.87 | 21.01 |
| Initial number of Jurkat cells in shaking cell culture with IFN-α |  |  | 14.06 | 14.39 | 13.09 |
| Initial number of Jurkat cells in static cell culture with IFN-α |  |  | 10.93 | 12.46 | 12.23 |
| Carrying capacity of Jurkat cells | $T_{max}$ | ${10}^{6}\times$cells/ml | 1.784 | | |
| NL4-3 (model 1) | | | | | |
| Growth rate of Jurkat cells in shaking cell culture without IFN-α | $g$ | day^-1^ | 0.4984 | | |
| Growth rate of Jurkat cells in static cell culture for without IFN-α |  |  | 0.4547 | | |
| Growth rate of Jurkat cells in shaking cell culture with IFN-α |  |  | 0.3840 | | |
| Growth rate of Jurkat cells in static cell culture with IFN-α |  |  | 0.4211 | | |
| Initial number of Jurkat cells in shaking cell culture for without IFN-α | $T(0)$ | ${10}^{4}\times$cells/ml | 17.59 | 19.30 | 21.40 |
| Initial number of Jurkat cells in static cell culture for without IFN-α |  |  | 20.31 | 20.26 | 22.79 |
| Initial number of Jurkat cells in shaking cell culture with IFN-α |  |  | 15.07 | 15.35 | 13.94 |
| Initial number of Jurkat cells in static cell culture with IFN-α |  |  | 11.55 | 13.27 | 13.12 |
| Carrying capacity of Jurkat cells in shaking cell culture without IFN-α | $T_{max}$ | ${10}^{6}\times$cells/ml | 1.764 | | |
| Carrying capacity of Jurkat cells in static cell culture for without IFN-α |  |  | 1.800 | | |
| Carrying capacity of Jurkat cells in shaking cell culture with IFN-α |  |  | 1.795 | | |
| Carrying capacity of Jurkat cells in static cell culture with IFN-α |  |  | 1.800 | | |
| CC and TF | | | | | |
| Growth rate of Jurkat cells in shaking cell culture without IFN-α | $g$ | day^-1^ | 0.3761 | | |
| Growth rate of Jurkat cells in static cell culture for without IFN-α |  |  | 0.4390 | | |
| Growth rate of Jurkat cells in shaking cell culture with IFN-α |  |  | 0.3209 | | |
| Growth rate of Jurkat cells in static cell culture with IFN-α |  |  | 0.3861 | | |
| Initial number of Jurkat cells in shaking cell culture for without IFN-α | $T(0)$ | ${10}^{4}\times$cells/ml | 10.68 | 9.688 | 10.74 |
| Initial number of Jurkat cells in static cell culture for without IFN-α |  |  | 9.009 | 9.774 | 9.457 |
| Initial number of Jurkat cells in shaking cell culture with IFN-α |  |  | 9.489 | 8.481 | 10.11 |
| Initial number of Jurkat cells in static cell culture with IFN-α |  |  | 9.108 | 8.850 | 9.598 |
| Carrying capacity of Jurkat cells | $T_{max}$ | ${10}^{6}\times$cells/ml | 1.989 | | |
