# Supplementary material for: Antithetic effect of interferon-α on cell-free and cell-to-cell HIV-1 infection
Source: PLoS Comput Biol. 2022 Apr 25;18(4):e1010053. doi: 10.1371/journal.pcbi.1010053 (PMC9037950; doi:10.1371/journal.pcbi.1010053)
Supplement: S3 Table — (DOCX) [file pcbi.1010053.s010.docx]

**S3 Table. The estimated initial values for HIV-1 strain NL4-3 by Model 0.**

| Variable | Symbol | Unit | Without IFN-α | | With IFN-α | |
| --- | --- | --- | --- | --- | --- | --- |
|  |  |  | Mean | 95% CI* | Mean | 95% CI* |
| Initial number of target cells in shaking cell culture | $T(0)$ | ${10}^{5}\times$cells/ml | 2.019 | 1.300 – 3.108 | 2.019 | 1.300 – 3.108 |
| Initial number of target cells in static cell culture |  |  | 1.413 | 0.9069 – 2.120 | 1.413 | 0.9069 – 2.120 |
| Initial number of infected cells in shaking cell culture | $I(0)$ | ${10}^{4}\times$cells/ml | 2.944 | 1.323 – 5.767 | 0.5676 | 0.2205 – 1.226 |
| Initial number of infected cells in static cell culture |  |  | 0.2059 | 0.08500 – 0.4165 | 0.08641 | 0.02185 – 0.2272 |
| Initial amount of HIV-1 in shaking cell culture | $V(0)$ | p24/ml | 10.30 | 5.277 –18.50 | 11.71 | 1.971 – 38.19 |
| Initial amount of HIV-1 in static cell culture |  |  | 7.172 | 2.930 – 14.69 | 6.450$\times{10}^{-4}$ | 9.569$\times{10}^{-6}$ – 3.908$\times{10}^{-3}$ |

*CI: credible interval.
