# Supplementary material for: Antithetic effect of interferon-α on cell-free and cell-to-cell HIV-1 infection
Source: PLoS Comput Biol. 2022 Apr 25;18(4):e1010053. doi: 10.1371/journal.pcbi.1010053 (PMC9037950; doi:10.1371/journal.pcbi.1010053)
Supplement: S4 Table — (DOCX) [file pcbi.1010053.s011.docx]

**S4 Table. The estimated initial values for HIV-1 strain NL4-3 by Model 1.**

| Variable | Symbol | Unit | Without IFN-α | | With IFN-α | |
| --- | --- | --- | --- | --- | --- | --- |
|  |  |  | Mean | 95% CI* | Mean | 95% CI* |
| Initial number of target cells in shaking cell culture | $T(0)$ | ${10}^{5}\times$cells/ml | 3.756 | 2.050 – 6.453 | 1.348 | 0.8612 – 2.016 |
| Initial number of target cells in static cell culture |  |  | 1.423 | 0.8332 – 2.316 | 1.111 | 0.7838 – 1.537 |
| Initial number of infected cells in shaking cell culture | $I(0)$ | ${10}^{4}\times$cells/ml | 1.714 | 0.7055 – 3.407 | 0.7309 | 0.4300 – 1.160 |
| Initial number of infected cells in static cell culture |  |  | 0.2532 | 0.1104 – 0.4901 | 0.08384 | 0.04331 – 0.1504 |
| Initial amount of HIV-1 in shaking cell culture | $V(0)$ | p24/ml | 2.014 | 1.600 – 2.478 | 0.1692 | 0.1387 – 0.2062 |
| Initial amount of HIV-1 in static cell culture |  |  | 0.5480 | 0.4637 – 0.6431 | 0.1708 | 0.07791 – 0.3201 |

*CI: credible interval.
