# Supplementary material for: Antithetic effect of interferon-α on cell-free and cell-to-cell HIV-1 infection
Source: PLoS Comput Biol. 2022 Apr 25;18(4):e1010053. doi: 10.1371/journal.pcbi.1010053 (PMC9037950; doi:10.1371/journal.pcbi.1010053)
Supplement: S8 Table — (DOCX) [file pcbi.1010053.s015.docx]

**S8 Table. The estimated initial values for HIV-1 strain CH077_CC by Model 1.**

| Variable | Symbol | Unit | Without IFN-α | | With IFN-α | |
| --- | --- | --- | --- | --- | --- | --- |
|  |  |  | Mean | 95% CI* | Mean | 95% CI* |
| Initial number of target cells in shaking cell culture | $T(0)$ | ${10}^{5}\times$cells/ml | 1.594 | 0.6026 – 3.240 | 1.594 | 0.6026 – 3.240 |
| Initial number of target cells in static cell culture |  |  | 2.751 | 0.7990 – 6.191 | 2.751 | 0.7990 – 6.191 |
| Initial number of infected cells in shaking cell culture | $I(0)$ | ${10}^{4}\times$cells/ml | 1.600 | 0.3039 – 5.272 | 0.3329 | 0.1035 – 0.8751 |
| Initial number of infected cells in static cell culture |  |  | 0.3340 | 0.03326 – 1.049 | 0.4545 | 0.1129 – 1.212 |
| Initial amount of HIV-1 in shaking cell culture | $V(0)$ | p24/ml | 1.136$\times{10}^{-5}$ | 3.024$\times{10}^{-6}$ – 3.100$\times{10}^{-5}$ | 753.3 | 356.2 – 1487 |
| Initial amount of HIV-1 in static cell culture |  |  | 4374 | 368.1 – 2.681$\times{10}^{5}$ | 1191 | 285.9 – 3026 |

*CI: credible interval.
