# Supplementary material for: Antithetic effect of interferon-α on cell-free and cell-to-cell HIV-1 infection
Source: PLoS Comput Biol. 2022 Apr 25;18(4):e1010053. doi: 10.1371/journal.pcbi.1010053 (PMC9037950; doi:10.1371/journal.pcbi.1010053)
Supplement: S9 Table — (DOCX) [file pcbi.1010053.s016.docx]

**S9 Table. The estimated initial values for HIV-1 strain CH077_TF by Model 1.**

| Variable | Symbol | Unit | Without IFN-α | | With IFN-α | |
| --- | --- | --- | --- | --- | --- | --- |
|  |  |  | Mean | 95% CI* | Mean | 95% CI* |
| Initial number of target cells in shaking cell culture | $T(0)$ | ${10}^{5}\times$cells/ml | 3.427 | 0.8168 – 8.741 | 3.427 | 0.8168 – 8.741 |
| Initial number of target cells in static cell culture |  |  | 3.008 | 1.261 – 6.280 | 3.008 | 1.261 – 6.280 |
| Initial number of infected cells in shaking cell culture | $I(0)$ | ${10}^{4}\times$cells/ml | 1.314 | 0.3103 – 3.488 | 0.3881 | 0.08198 – 1.083 |
| Initial number of infected cells in static cell culture |  |  | 0.2250 | 0.05859 – 0.6255 | 0.3083 | 0.02585 – 1.213 |
| Initial amount of HIV-1 in shaking cell culture | $V(0)$ | p24/ml | 58.88 | 7.667 – 227.3 | 6.867 | 1.058 – 21.48 |
| Initial amount of HIV-1 in static cell culture |  |  | 52.00 | 14.22 – 52.192 | 3874 | 142.6 – 1.881$\times{10}^{5}$ |

*CI: credible interval.
